# Selenium Nanoparticles Selectively Target KRAS G13D to Inhibit Colorectal Cancer

**DOI:** 10.1002/advs.202523795

**Published:** 2026-08-18

**Authors:** Xiaoting Liu, Chong Wu, Shiyao Song, Li Ma, Shaoqing Huang, Yuting He, Qinghai Li, Yanzhou Chang, Dan Jiang, Yong Mei, Junchao Cai, Zhenshuang Du, Tianfeng Chen, Weiling He

**Affiliations:** ^1^ Department of Gastrointestinal Surgery School of Medicine Xiang'an Hospital of Xiamen University Xiamen University Xiamen Fujian China; ^2^ Department of Gastrointestinal Surgery The First Affiliated Hospital Sun Yat‐sen University Guangzhou Guangdong China; ^3^ Department of Chemistry College of Chemistry and Materials Science Jinan University Guangzhou China; ^4^ Department of Pediatrics The First Affiliated Hospital Sun Yat‐sen University Guangzhou Guangdong China; ^5^ Department of Immunology Zhongshan School of Medicine Sun Yat‐sen University Guangzhou Guangdong China

**Keywords:** colorectal cancer, GPX2, KRAS mutant, nanoparticles, selenium metabolism

## Abstract

Despite substantial progress in KRAS‐targeted therapies, specific inhibitors of the KRAS G13D mutant remain unavailable. In a retrospective analysis, we found a notable downregulation of glutathione peroxidase 2 (GPX2) in KRAS G13D‐ mutant colorectal cancer (CRC), which correlated with poor outcomes. We hypothesized that GPX2 acts as a tumor suppressor and that its restoration could render KRAS G13D‐driven tumors vulnerable. In this study, we developed a selenium nanoparticle (SeNPs)‐based nanostrategy to suppress KRAS G13D‐driven tumors by modulating GPX2 expression. SeNPs upregulated GPX2 expression, thereby inhibiting metastasis through the GPX2‐HIF1α‐VEGF axis. Moreover, SeNPs release selenite (SeO_3_^2−^) in situ, which interacts with the mutant pocket (residues 13–17) of the KRAS G13D protein. In vivo, this multimodal strategy demonstrated exceptional efficacy. In orthotopic CRC models, SeNPs significantly inhibited primary tumor growth and effectively prevented liver metastasis. In cell‑derived xenograft (CDX) and patient‐derived xenograft (PDX) models, SeNPs achieved tumor inhibition rates of 70% and 61%, respectively. Collectively, this study demonstrates the first‐in‐class therapeutic potential of SeNPs against this recalcitrant KRAS mutant and provides a novel nanoplatform for KRAS G13D‐targeted therapy.

## Graphical abstract

The mechanisms of SeNPs therapy in cancer treatment, encompass three parallel actions: (1) seleno‐amino acids, key metabolites, upregulate GPX2 expression, thereby inhibiting tumor metastasis via the GPX2‐HIF1α‐VEGF signaling pathway; (2) selenite (SeO_3_^2−^), an inorganic metabolite, forms hydrogen bonds with amino acid residues 13–17 of the KRAS G13D‐ mutant protein, thereby disrupting oxidative phosphorylation and ultimately inhibiting tumor progression in KRAS G13D mutant cancers; (3) SeO_3_^2−^ induces apoptosis via the reactive oxygen species pathway.

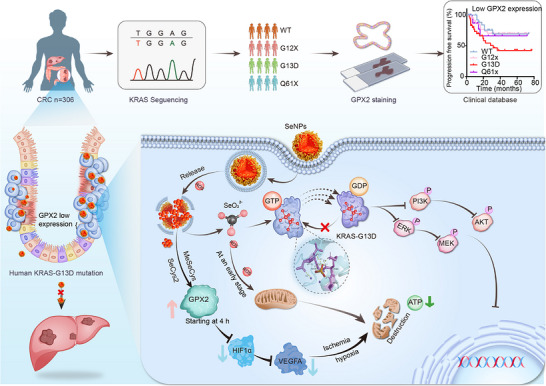

## 1 Introduction

Colorectal cancer (CRC) is the second most common cause of cancer‐related death worldwide [1, 2, 3]. Approximately 50% of patients with CRC harbor KRAS mutations [4, 5, 6, 7]. KRAS, a small guanosine triphosphatase (GTPase), regulates cell growth and differentiation through GTP binding and hydrolysis under normal conditions [8, 9, 10]. Furthermore, it plays a crucial role in multiple signaling pathways, such as RAS‐MAPK, PI3K‐Akt, and JAK‐STAT [6, 7, 11]. Mutations in KRAS are predominant at codons 12, 13, and 61 within its GTPase domain, leading to its continuous activation, thereby driving cancer progression [12, 13]. It can mediate cancer resistance and ultimately influence treatment decisions [14, 15]. For example, the presence of KRAS mutations in CRC predicts a lack of clinical benefit from Epidermal Growth Factor Receptor (EGFR)‐targeted monoclonal antibodies, such as cetuximab and panitumumab [16, 17, 18, 19]. Effective inhibitors have not yet been identified for most oncogenic KRAS mutants. Overall, very limited effective therapeutic options are available to patients with CRC harboring KRAS mutations who develop recurrent disease.

The G13D mutation in KRAS occurs in the GTP/GDP‐binding pocket. In this mutation, the small nonpolar Gly at position 13 is replaced by the bulkier, negatively charged Asp [17, 20]. This substitution introduces a significant negative charge at the P‐loop region, profoundly altering the local electrostatic environment [21, 22, 23]. Consequently, the exchange rate between GDP and GTP increases. Furthermore, this mutation impairs the intrinsic GTP hydrolysis rate [24, 25, 26]. Together, these effects promote a more frequent replacement of GDP by GTP, resulting in constitutive activation of the KRAS protein. Notably, compared with codon 12 mutations, codon 13 mutations—especially G13D—display weaker transforming activity. The unique molecular landscape of the KRAS G13D variant underlies the enhanced tumor aggressiveness noted in patients harboring this mutation; moreover, it also contributes to its resistance to effective therapeutic targeting.

The KRAS G13D mutation is predominantly observed in gastrointestinal tumors [21, 27, 28]. It represents the third most common KRAS mutation in CRC, occurring in approximately 8–19% of patients with CRC [17, 20, 29]. Furthermore, compared with CRCs with other KRAS mutations, those with the G13D mutation exhibit greater aggressiveness [30, 31]. These tumors are often characterized by local invasion or distant metastasis at the time of diagnosis [32]. Patients harboring the KRAS G13D mutation typically respond poorly to standard chemotherapy and have a poorer prognosis than those harboring other KRAS mutations [33, 34]. Although some studies have suggested a potential sensitivity to EGFR inhibitors, the clinical benefit in KRAS G13D‐mutant tumors remains unclear [16, 35, 36]. Monotherapy with MEK inhibitors often results in acquired resistance. This resistance arises from the high affinity of KRAS G13D to RAF, which promotes sustained MEK phosphorylation and bypasses MEK inhibition. Additionally, KRAS G13D exhibits a high level of intrinsic activity, which greatly reduces its dependence on guanine nucleotide exchange factors, thereby limiting the efficacy of SOS1 inhibitors. Pan‐RAS (ON) tri‐complex inhibitors, such as daraxonrasib (RMC‐6236), which are now in Phase 3 development, have shown activity across multiple KRAS alleles, including G12X, G13X, and Q61X [37, 38]; pan‐KRAS (OFF) compounds, such as BI‐2865, bind KRAS G13D with nanomolar affinity (KD approximately 4.3 nM) and have been co‐crystallized with this variant [39]. Next‐generation candidates, including LY4066434, have entered Phase 1 trials with G13D explicitly included in eligibility criteria (NCT06607185). However, broad RAS coverage also results in the simultaneous inhibition of wild‐type (WT) RAS, thereby narrowing the therapeutic window, and clinical efficacy in KRAS G13D‐mutant CRC has not been established. To date, no mutation‐specific therapies have been approved for KRAS G13D‐mutant CRC. Given the unique biological features of this mutation, the development of targeted treatment strategies is essential for advancing precision medicine for CRC.

Glutathione peroxidase 2 (GPX2) is primarily expressed in the gastrointestinal tract and is critical for maintaining mucosal homeostasis [40, 41]. Its functions in the intestine are highly specific and cannot be fully compensated by other selenoproteins, including members of the same family [42, 43]. During the development and progression of CRC, GPX2 exerts complex regulatory functions. Studies have shown that the loss of GPX2 promotes the initiation and progression of CRC and is associated with increased tumor malignancy [40, 44, 45, 46, 47]. However, the role of GPX2 appears to be context‐ and stage‐dependent. In some CRC tissues, GPX2 upregulation may reflect the inherently high expression patterns observed in intestinal epithelial cells [47, 48]. Although GPX2 knockdown leads to a significant reduction in CRC cell proliferation, it simultaneously enhances their invasive and migratory abilities. Therefore, GPX2 may inhibit tumor cell invasion and migration while potentially promoting proliferation [49]. This suggests that GPX2 functions in a spatiotemporally dependent manner in CRC. In the early stages, it facilitates tumor cell proliferation by alleviating oxidative stress and maintaining intracellular homeostasis. However, in the later stages, it suppresses tumor invasion and migration by modulating specific signaling pathways [50, 51]. Notably, GPX2 expression is frequently downregulated in tumors with a high mutational burden, suggesting its involvement in CRC progression [52]. Selenium (Se) is an essential trace element required for the synthesis of selenoproteins such as GPX2 [53, 54, 55, 56]. Its dietary availability directly influences selenoprotein expression and activity, thereby modulating oxidative stress, inflammation, and key signaling pathways involved in CRC progression [57]. Epidemiological evidence has linked Se deficiency to increased cancer risk, particularly in individuals with low systemic Se reserves [58, 59, 60]. Se exerts anticancer effects through epigenetic modulation and direct regulation of oncogenic signaling [61, 62, 63]. GPX2, a gastrointestinal selenoenzyme, is pivotal for maintaining epithelial homeostasis and limiting tumor progression [46, 64, 65, 66]. Biological activity of Se is largely mediated by its incorporation into selenoproteins [67, 68]. In addition, Se can be metabolized into inorganic forms—selenite (SeO_3_^2−^) and selenate (SeO_4_^2−^)—which possess antitumor properties. Structurally, SeO_3_^2−^ mimics phosphate groups (PO_4_^3−^) that bind to the KRAS P‐loop; however, its lower charge density relative to phosphate may result in a distinct binding pattern within the pocket compared with the native phosphate groups. However, the clinical application of sodium SeO_3_^2−^ is limited by its poor tumor selectivity and systemic bioavailability. Traditional organic Se compounds also exhibit low solubility and efficacy [69, 70, 71, 72, 73, 74]. In contrast, selenium nanoparticles (SeNPs) represent a promising class of elemental Se formulations with superior bioavailability, lower toxicity, and enhanced antioxidant and antitumor activities compared with inorganic and organic Se [75, 76, 77, 78, 79, 80].

In this study, we investigated the association between GPX2 expression and the progression of CRC harboring the KRAS G13D mutation and evaluated the therapeutic potential of SeNPs. A retrospective analysis of 306 CRC tumor samples, including wild‐type KRAS and various KRAS‐mutant subtypes, revealed downregulation of GPX2 expression in KRAS G13D‐mutant CRC, which was correlated with poor prognosis. Based on this finding, we designed and synthesized surface‐modified, Se‐rich SeNPs. The antitumor efficacy of the SeNPs was systematically validated using in vitro assays and multiple in vivo models. Remarkably, SeNPs eliminated liver metastasis in an orthotopic CRC model and demonstrated potent antitumor efficacy, with tumor inhibition rates of 70% and 61% in the cell‑derived xenograft (CDX) and patient‐derived xenograft (PDX) models, respectively. Mechanistically, SeNPs exerted their antitumor effects by upregulating GPX2 expression through both Delta N‐terminal p63 (ΔNp63) signaling and selenoamino acid metabolism. This upregulation inhibited tumor progression via the GPX2‐HIF1α‐VEGF signaling axis. In parallel, SeNPs activated the reactive oxygen species (ROS) pathway, triggering mitochondrial damage and apoptosis. Moreover, molecular docking and functional assays demonstrated that SeNPs are metabolized into SeO_3_^2−^, which binds to residues 13–17 in the P‐loop of the KRAS G13D protein, thereby suppressing downstream ERK and Akt signaling. Collectively, our findings establish GPX2 as a potential therapeutic target in KRAS G13D‐mutant CRC and demonstrate that engineered SeNPs can effectively restore GPX2 expression. We provide compelling evidence that SeNPs constitute a promising precision nanotherapeutic strategy for patients with KRAS G13D‐mutant CRC.

## 2 Results

### 2.1 GPX2 Downregulation in CRC Harboring the KRAS G13D Mutation is Associated With Poor Prognosis

**FIGURE 1:**
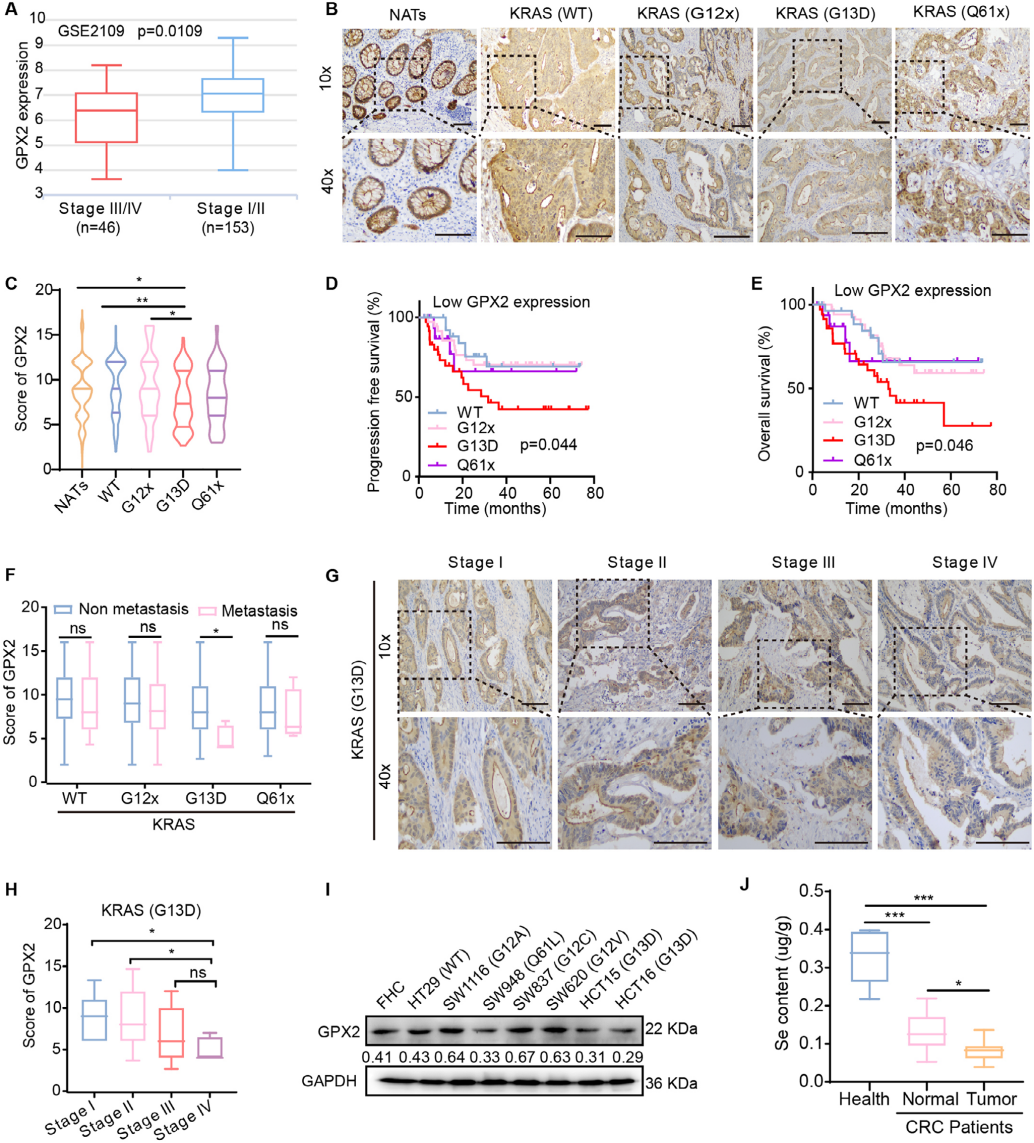
Low expression of GPX2 is associated with poor prognosis in CRC harboring the G13D mutation. (A) Comparison of GPX2 expression levels between early‐ and advanced‐stage CRC samples in the GSE2109 dataset. (B) Representative images and (C) expression scores of GPX2 in normal intestinal mucosa and CRC tissues with different KRAS mutation statuses (*n* = 306). Scale bar: 50 µm. (D) Kaplan‐Meier curves for PFS and (E) OS comparing different KRAS mutation subtypes within the GPX2‐low expression group. (F) The expression of GPX2 in metastatic and non‐metastatic CRC patients with KRAS mutation. (G) Representative images and (H) quantification of GPX2 expression in KRAS G13D‐ mutant CRC tissues across different stages. Scale bar: 50 µm. (I) Western blot analysis of GPX2 expression in normal intestinal epithelial cells, KRAS wild‐type CRC cells, and CRC cell lines harboring different KRAS mutations. (J) Se levels in the intestinal epithelium of healthy individuals, in the normal‐adjacent tissues, and matched tumors of KRAS G13D mutation patients. Statistical significance was assessed using Mann–Whitney (C, F, H, and J) or the log‐rank (Mantel–Cox) test (D and E). ns, non‐significant, ^*^ *p* < 0.05, ^**^ *p* < 0.01, ^***^ *p* < 0.001.

GPX2 is a Se‐dependent selenoprotein predominantly expressed in the intestinal epithelium [40, 41, 45]. Se deficiency suppresses GPX2 expression and is associated with an increased CRC incidence [46, 58, 64, 65]. GPX2‐knockout mice have been demonstrated to be more prone to spontaneous intestinal tumor development [40, 44, 45]. In addition, GPX2 expression was negatively correlated with tumor mutational burden (TMB) in Colon Adenocarcinoma [52]. To evaluate the clinical significance and potential biological role of GPX2 in KRAS‐mutant CRC, we first performed a Kaplan–Meier survival analysis using the KM‐Plotter database, which showed that low GPX2 expression was associated with poor progression‐free survival (PFS), relapse‐free survival, and overall survival (OS) (Figure S1A–C). Subsequently, we analyzed *GPX2* mRNA expression in CRC using data from The Cancer Genome Atlas (TCGA). GPX2 expression was found to be upregulated in tumor tissues compared with adjacent normal epithelium (Figure S2A). However, the lncAR platform revealed decreased GPX2 expression in advanced‐stage tumors and metastatic CRC (Figure 1A and Figure S2B), suggesting stage‐ or context‐dependent regulation of GPX2 during CRC progression. We hypothesized that reduced GPX2 expression promotes CRC progression and is correlated with poor clinical outcomes. We evaluated GPX2 protein expression and its clinical relevance in 306 CRC specimens harboring various KRAS mutations. The association between GPX2 expression and clinicopathological features is summarized in Table S2. GPX2 expression progressively decreased with advancing tumor stage (Figure S2C), consistent with bioinformatic analyses. Notably, approximately 50% of patients with CRC harbor KRAS mutations [4, 5, 6, 7], and GPX2 expression is inversely correlated with TMB [81]. Next, we analyzed GPX2 expression across tumors harboring different KRAS mutations. GPX2 expression was reduced in tumors harboring the KRAS G13D mutation compared with that in normal intestinal epithelium, KRAS wild‐type tumors, and those with G12X or Q61X mutations (Figure 1B,C). Further survival analysis demonstrated that reduced GPX2 expression was correlated with poorer PFS and OS, most notably in KRAS G13D‐mutant CRC, which exhibited the poorest prognostic outcomes (Figure 1D,E and Figure S3A,B). The KM‐Plotter analysis corroborated our findings, further supporting the association between reduced GPX2 expression and poor prognosis in CRC. Further stratification revealed that GPX2 expression was lower in patients with KRAS G13D‐ mutant CRC having distant metastases than in those without metastases. In contrast, this trend was not observed in the KRAS WT, G12X, or Q61X groups.

The KRAS G13D mutation predominantly occurs in gastrointestinal tumors and is the third most common KRAS mutation in CRC [12]. CRC harboring the KRAS G13D mutation is considered more aggressive than other KRAS variants and frequently presents with local invasion or distant metastasis at diagnosis [82]. To further evaluate the relationship between GPX2 expression and KRAS G13D‐mutant CRC, we examined GPX2 levels in clinical tumor samples. Our analysis revealed a significant inverse correlation between GPX2 expression and tumor‐node‐metastasis stage in patients with KRAS G13D‐mutant CRC (Figure 1F); specifically, GPX2 expression was markedly lower in advanced‐stage tumors (Figure 1G,H). To further validate GPX2 expression across distinct KRAS mutation backgrounds, we measured GPX2 protein levels in normal intestinal epithelial cells (FHC), WT KRAS CRC cells, and multiple KRAS‐mutant CRC cell lines. Consistent with clinical findings, GPX2 expression was markedly reduced in cell lines harboring the KRAS G13D mutation compared with that in both normal colonic epithelial cells and other KRAS‐mutant or WT cell lines (Figure 1I and Figure S4A). These results confirm the selective downregulation of GPX2 in KRAS G13D‐mutant CRC. Epidemiological studies have indicated an inverse relationship between Se intake and CRC incidence, and Se is known to regulate the expression of selenoproteins such as GPX2 [46, 58, 64, 65]. Based on these findings, we hypothesized that reduced GPX2 expression in KRAS G13D‐mutant tumors may be related to Se deficiency. To test this hypothesis, we performed inductively coupled plasma mass spectrometry to quantify Se content in fresh tissues collected from nine healthy individuals and nine patients with CRC harboring the KRAS G13D mutation. The samples included tumor tissues and matched adjacent normal mucosa. The results revealed that Se concentrations were lower in the KRAS G13D‐mutant CRC tumor tissues, indicating a potential Se‐deficient state (Figure 1J). Taken together, our findings demonstrate that GPX2 is selectively downregulated in KRAS G13D‐mutant CRC.

### 2.2 SeNPs Inhibit the Progression of CRC in Vitro

**FIGURE 2:**
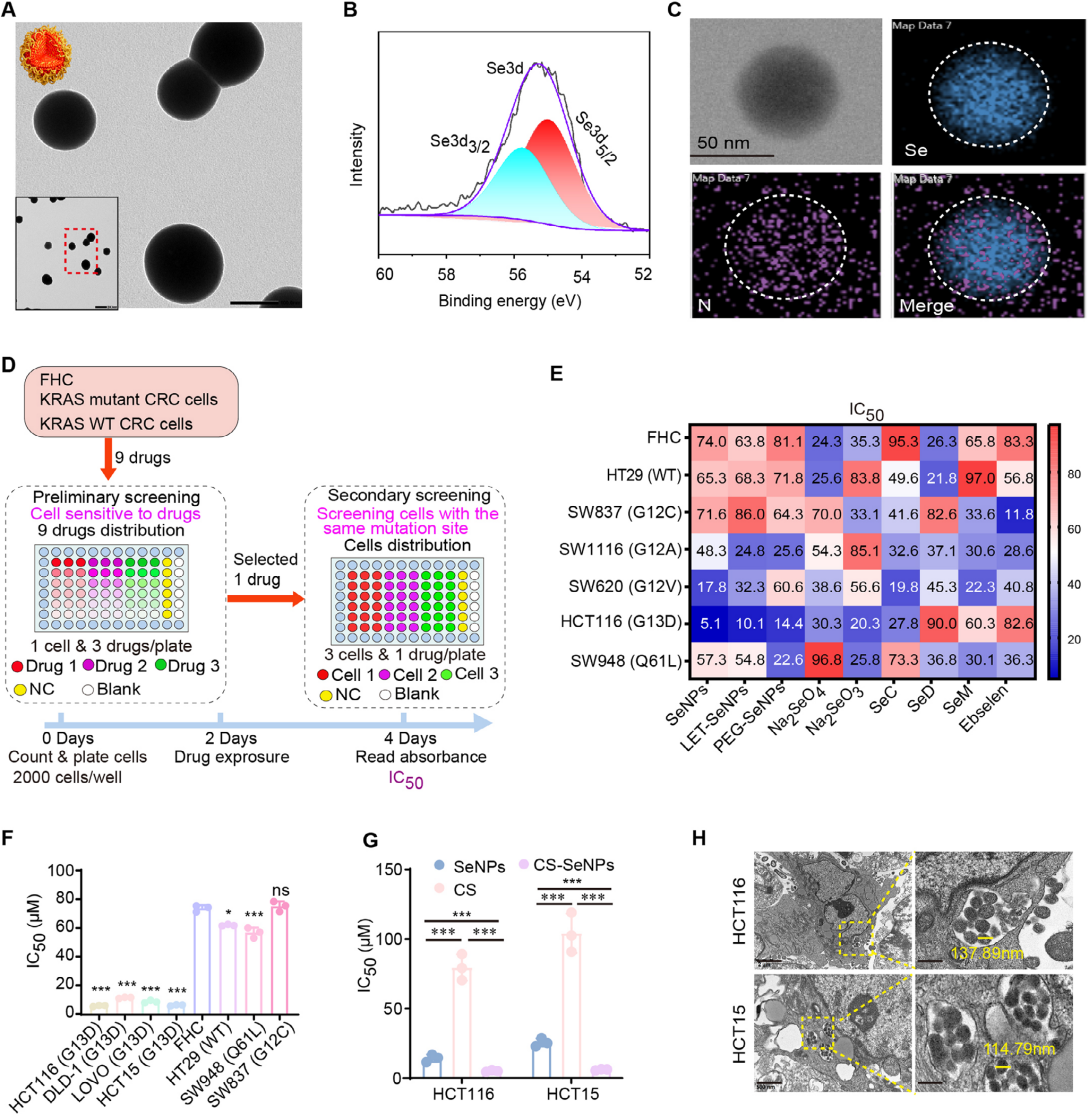
The antitumor efficacy of SeNPs on CRC in vitro*. (*A) Schematic diagram and TEM image of SeNPs. (B) High‐resolution X‐ray photoelectron spectroscopy (XPS) spectrum of SeNPs. (C) EDS mapping of SeNPs showing the elemental distribution. (D) The drug screening process schematic is as follows: FHC, HT29 (KRAS WT), and KRAS mutant cells are seeded in a 96‐well plate. 9 types of Se‐containing drugs are incubated for the preliminary screening. A drug that demonstrates significant antitumor effects was selected for secondary screening. (E) Heatmap of raw IC50 values (µM, unscaled). Lower IC50 indicates higher sensitivity (shown in blue; *n* = 3). (F) IC_50_ values of SeNPs in different KRAS mutant CRC cell lines after 48 h treatment, IC50 values were log_10_ transformed before analysis, normal intestinal epithelial cells (FHC) as the reference control (Column 5). (G) IC_50_ values of different drugs in KRAS G13D‐mutant CRC cells following 48 h treatment. IC50 values were log_10_ transformed before analysis. (H) TEM images of ultrastructural changes in cells incubated with SeNPs in HCT116 and HCT15 cells (5 µM, 48 h). Yellow arrows indicate SeNPs. Statistical significance was analyzed and calculated using one‐way ANOVA (F), or by two‐way ANOVA (G), ns, non‐significant, ^*^ *p* < 0.05, ^**^ *p* < 0.01, ^***^ *p* < 0.001.

GPX2 expression is markedly downregulated, and Se levels are deficient in patients with CRC harboring the KRAS G13D mutation. Se exists in organic, inorganic, or nanoparticulate forms. Compared with traditional organic and inorganic Se compounds, nanosized Se exhibits higher bioavailability, improved antitumor activity, and lower toxicity [61, 63, 83]. To investigate the therapeutic potential of nano‐sized Se as a Se‐supplementing nanomedicine in patients with KRAS G13D mutations and low GPX2 expression, we synthesized SeNPs with a high Se content [63, 84]. Transmission electron microscopy (TEM) analysis showed that the SeNPs were uniformly dispersed, with an average particle size of approximately 150 nm (Figure 2A). Dynamic light scattering showed that the SeNPs had a particle diameter of 152.3 nm and a polydispersity index of 0.092 (Figure S5A). Zeta potential analysis revealed that the surface of SeNPs is approximately electrically neutral (zeta potential of +1.5 mV). This helps avoid rapid clearance by the immune system (Figure S5B). Fourier‐transform infrared spectroscopy analysis displayed a characteristic absorption peak at 780 cm^−1^, attributed to C–Se stretching vibrations (Figure S5C). X‐ray photoelectron spectroscopy demonstrated distinct peaks for C1s, O1s, and Se3d (Figure S5D). The high‐resolution Se spectrum showed peaks at 3d5/2 and 3d3/2, with energies of 54.9 and 55.7 eV, respectively, which confirmed the presence of Se in the SeNPs in its zero‐valent state (Figure 2B and Figure S5E,F). Furthermore, elemental mapping analysis of energy‐dispersive X‐ray spectroscopy revealed the presence of Se and nitrogen within the nanomaterials, validating the effective integration of these elements into the chitosan‐coated SeNPs (Figure 2C). The SeNPs maintained a relatively stable hydrated particle size over an extended period in these solutions (Figure S5G).

To assess the therapeutic potential of SeNPs in KRAS G13D‐mutant CRC with low GPX2 expression, we performed a comprehensive drug sensitivity screening. A total of nine Se‐containing compounds were evaluated, including the high‐Se‐content SeNPs synthesized by us, along with eight reference compounds: two nano‐Se formulations (Lentinan‐modified SeNPs and PEGylated SeNPs), two inorganic forms (Na_2_SeO_3_ and Na_2_SeO_4_), and four organoselenium compounds (Ebselen, 4‐nitrophenylselenadiazole, selenocysteine, and selenomethionine). A two‐tiered screening strategy was used in this study. In the primary screening, drug responses were profiled across seven cell lines, including normal intestinal epithelial cells (FHC), KRAS WT CRC cells (HT29), and CRC cells with distinct KRAS mutations. Cells were seeded in 96‐well plates (2000 cells/well), treated with a six‐point concentration gradient (3.125–100 µM), and incubated for 48 h. The secondary screening evaluated the reproducibility of antitumor activity in additional CRC cell lines with the same KRAS mutation (Figure 2D). Among all compounds tested, the greatest selective cytotoxicity against KRAS G13D‐mutant CRC cells was demonstrated by SeNPs (Figure 2E,F). Mechanistic studies confirmed that the Se core of the SeNPs was essential for this effect (Figure 2G). Functional assays showed that SeNPs impaired the proliferation, migration, and invasion of KRAS G13D‐mutant CRC cells (Figure S6A–E). TEM revealed that the SeNPs entered the cells via membrane engulfment and endocytosis (Figure 2H). Together, these results demonstrate that the engineered SeNPs constructed in this study exhibit robust antitumor activity against KRAS G13D‐mutant CRC cells, supporting their potential as targeted Se‐based nanotherapeutics.

### 2.3 The Inhibitory Mechanism of SeNPs of KRAS G13D‐Mutant CRC

**FIGURE 3:**
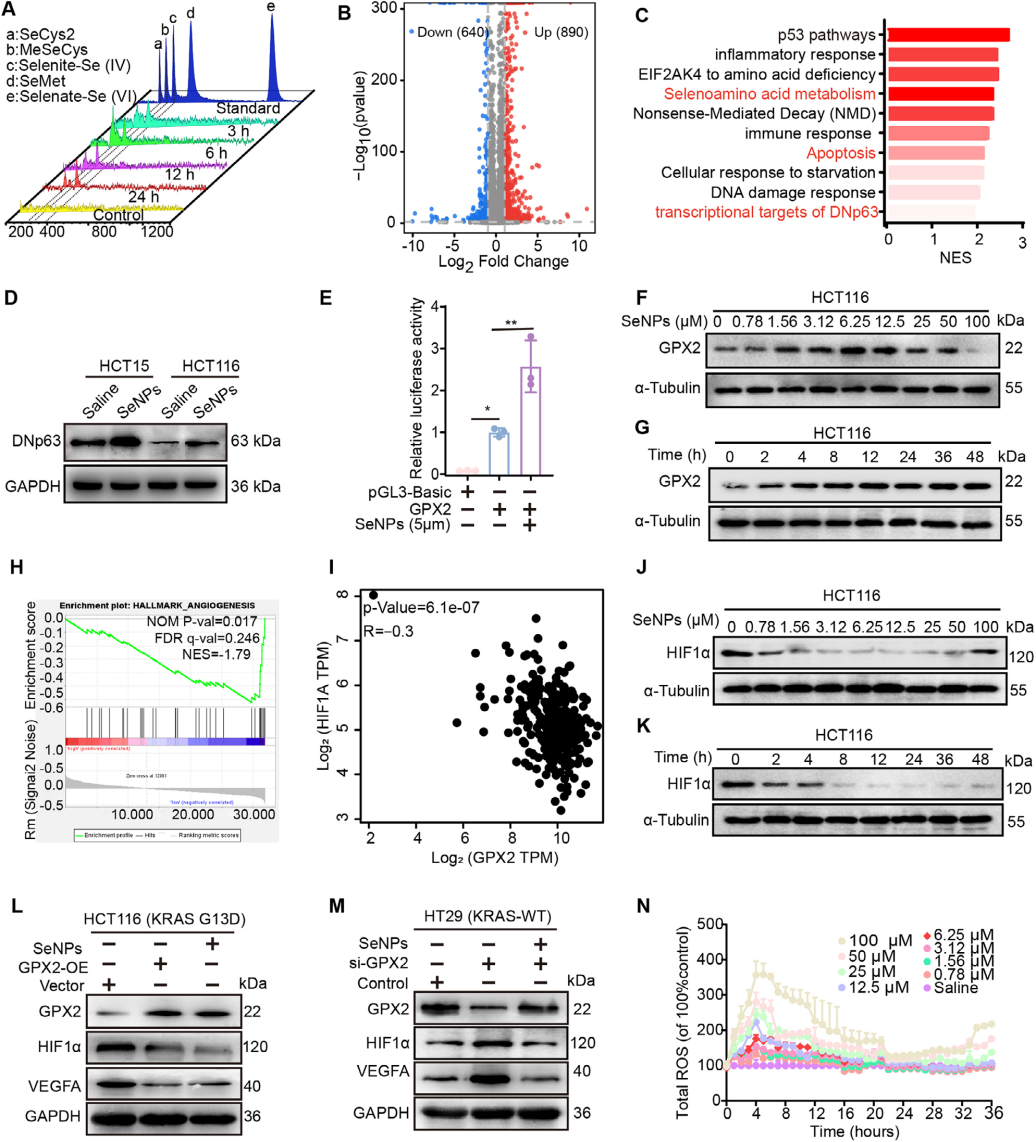
SeNPs inhibit CRC via the HIF1α‐VEGFA axis. (A) Intracellular selenium metabolites in HCT116 cells (5 µM) at the indicated time points analyzed by HPLC‐ICP‐MS; peaks: a, SeCys2; b, MeSeCys; c, Selenite (SeO_3_^2−^)‐Se (IV); d, SeMet; e, Selenate‐Se (VI). (B) Volcano plot of differentially expressed genes between SeNPs‐treated and control HCT116 cells (5 µM, 48 h). (C) Top 10 significantly enriched pathways from GSEA of the data in (B). (D) Western blot analysis of ΔNp63 protein expression following SeNPs treatment in HCT15 and HCT116 cells (5 µM, 48 h). (E) Dual‐luciferase reporter assay in the HCT116 cells evaluating ΔNp63‐mediated transcriptional activation of the GPX2 promoter. Cells were transfected for 24 h prior (GPX2 promoter: 300 ng, ΔNp63 expression plasmid: 300 ng, control plasmid: 50 ng) to SeNPs exposure (5 µM, 24 h). (*n* = 3). (F,G) Western blot analysis of GPX2 expression in response to SeNPs treatment at varying concentrations (F) and time points (G) (*n* = 3). (H) GSEA enrichment plot of angiogenesis‐related gene sets. (I) Correlation analysis between GPX2 and HIF1α mRNA expression levels. (J) Western blot analysis of HIF1α expression following treatment with different concentrations of SeNPs (*n* = 3). (K) Western blot analysis of HIF1α expression at different time points after SeNPs treatment (*n* = 3). (L) Western blot analysis of HIF1α and VEGFA protein levels following either GPX2 overexpression (2 µg, 48 h) or SeNPs treatment (5 µM, 48 h). (M) Western blot analysis of HIF1α and VEGFA expression following GPX2 knockdown (siRNA, 50 nM) with or without SeNPs treatment (5 µM, 48 h). Cells were transfected for 48 h before exposure to SeNPs. (N) Time‐course analysis of intracellular ROS levels in HCT116 cell line following treatment with SeNPs at various concentrations (*n* = 3). GAPDH or α‐tubulin was used as a loading control. Statistical significance was analyzed by one‐way ANOVA, ^*^ *p* < 0.05, ^**^ *p* < 0.01.

To develop a thorough understanding of the antitumor activity of SeNPs, we first confirmed their efficient internalization into KRAS G13D‐mutant CRC cells (Figure S7A). Further evaluation of Se biotransformation in these mutant cells revealed that, after 3 h of SeNPs treatment, the levels of selenocystine (SeCys_2_), methylselenocysteine (MeSeCys), and SeO_3_^2−^ markedly increased. With prolonged incubation (12 and 24 h), SeCys_2_ and MeSeCys levels progressively declined, suggesting the ongoing metabolic transformation of SeNPs, whereas SeO_3_^2−^ levels declined at a slower rate (Figure 3A and Figure S7B).

To investigate the molecular mechanisms mediated by SeNPs in KRAS G13D‐mutant CRC cells, we performed transcriptome profiling following SeNPs treatment. RNA‐seq analysis revealed that SeNPs altered the expression of 1530 genes, with 890 upregulated and 640 downregulated genes (Figure 3B). Pathway enrichment analysis indicated that the upregulated genes were primarily associated with the p53 signaling pathway, selenoprotein metabolism, apoptosis, and ΔNp63 signaling axis (Figure 3C). Western blot analysis further confirmed that SeNPs treatment increased ΔNp63 protein expression (Figure 3D). ΔNp63 is a key transcriptional activator regulating GPX2 expression [85]. Consistently, dual‐luciferase reporter and quantitative polymerase chain reaction analyses demonstrated that SeNPs enhanced GPX2 promoter activity and upregulated its mRNA expression (Figure 3E and Figure S7C), suggesting that SeNPs enhance ΔNp63‐mediated transcriptional activation of GPX2. In addition, selenoamino acids generated from SeNPs metabolism may contribute to the biosynthesis of selenoproteins. Treatment of KRAS G13D‐ mutant cells with varying concentrations of SeNPs revealed that low concentrations significantly upregulated GPX2 expression, whereas high concentrations suppressed GPX2 expression (Figure 3F). Time‐course experiments using low concentrations of SeNPs showed that GPX2 expression was not inhibited by prolonged exposure (Figure 3G). To further explore the molecular mechanisms by which SeNPs inhibit CRC progression through GPX2, we first performed gene set enrichment analysis (GSEA), which revealed a negative correlation between GPX2 expression and angiogenesis (Figure 3H), suggesting that GPX2 may suppress tumor progression by inhibiting angiogenesis. Vascular endothelial growth factor A (VEGFA) is a key regulator of angiogenesis, and its transcription is primarily controlled by hypoxia‐inducible factor 1‐alpha (HIF1α) [86]. Based on these findings, we hypothesized that GPX2 may inhibit malignant progression in KRAS G13D‐mutant CRC cells by modulating the HIF1α–VEGFA signaling axis. Analysis of the Gene Expression Profiling Interactive Analysis (GEPIA) database revealed a negative correlation between GPX2 and HIF1α expression (Figure 3I and Figure S8A,B). Subsequent experiments showed that low concentrations of SeNPs suppressed HIF1α expression, whereas high concentrations stabilized HIF1α (Figure 3J), and prolonged treatment with low concentrations maintained sustained suppression of HIF1α (Figure 3K). Further validation of GPX2‐mediated regulation demonstrated that both SeNPs treatment and GPX2 overexpression significantly reduced HIF1α and VEGFA protein levels (Figure 3L). In CRC cells with high endogenous GPX2 expression, GPX2 knockdown resulted in upregulation of HIF1α and VEGFA, which was effectively reversed by SeNPs treatment (Figure 3M). These findings suggest that GPX2 suppresses the HIF1α–VEGFA axis.

SeO_3_^2−^ is known to induce the production of ROS in cells. ROS‐mediated DNA damage initiates mitochondrial apoptosis [87, 88, 89]. KRAS G13D‐mutant CRC cells are sensitive to oxidative phosphorylation (OXPHOS) and mitochondrial translation inhibitors [90, 91, 92, 93, 94]. To elucidate the dynamic relationship between SeNPs‐induced ROS generation and the GPX2 antioxidant system, we measured ROS levels at different time points and different SeNPs concentrations. SeNPs treatment was found to rapidly induce ROS production in the early stages, consistent with the rapid generation of SeO_3_^2−^. After 4 h of treatment, GPX2 protein expression was significantly upregulated, accompanied by a decline in ROS levels, which remained relatively low under treatment with a low SeNPs concentration. However, under treatment with a high concentration of SeNPs, GPX2 expression was suppressed, leading to secondary accumulation of ROS (Figure 3N). This biphasic response suggests that SeNPs initially induce a SeO_3_^2−^‐mediated ROS burst to trigger apoptotic signaling, followed by the upregulation of GPX2 as a compensatory antioxidant mechanism.

Consistently, RNA sequencing analysis showed that SeNPs treatment downregulated genes enriched in mitochondrial OXPHOS and calcium signaling pathways (Figure S9A), whereas GSEA using the TCGA dataset revealed a negative correlation between GPX2 expression and OXPHOS activity (Figure S9B). Consistent with these findings, the expression of multiple genes involved in mitochondrial respiration and biogenesis was suppressed by SeNPs treatment (Figure S9C). Morphological observations further demonstrated that SeNPs disrupted mitochondrial integrity, as evidenced by network fragmentation and structural damage, whereas control cells maintained a typical filamentous mitochondrial architecture (Figure S9D,E). Functionally, SeNPs treatment induced apoptosis in KRAS G13D‐mutant CRC cells and markedly reduced ATP production (Figure S9F–H).

### 2.4 SeO_3_^2−^ Targets the P‐Loop to Disrupt KRAS G13D GTPase Activity Competitively

**FIGURE 4:**
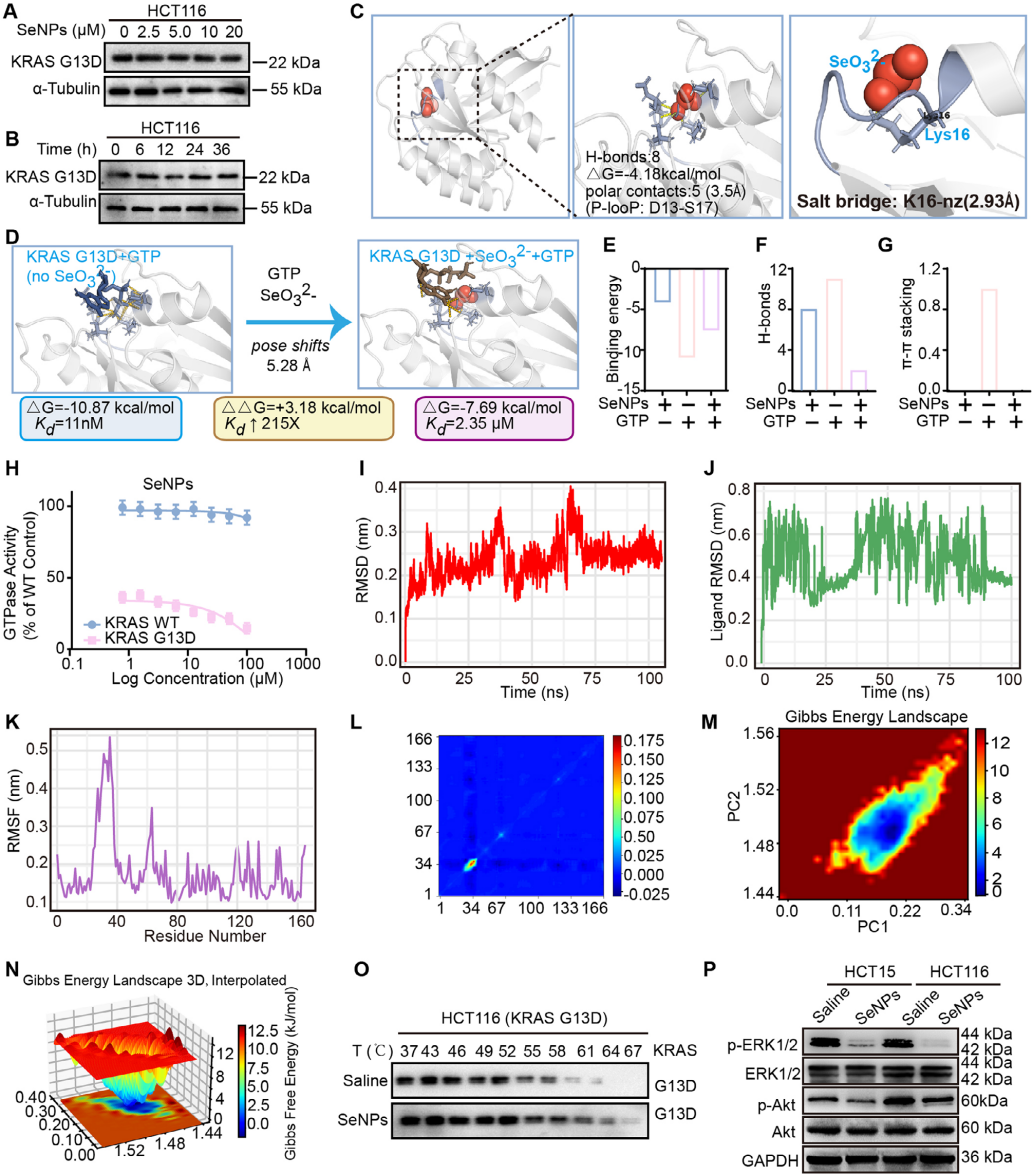
The SeO_3_^2−^ competitively disrupts GTP binding and hydrolysis of KRAS G13D. (A) Western blot analysis of KRAS G13D protein expression in cells treated with increasing concentrations of SeNPs (*n* = 3). (B) Western blot analysis of KRAS G13D expression levels in HCT116 cells at the indicated time points after exposure to 5 µM SeNPs. (*n* = 3). (C) Molecular docking simulation (AutoDock Vina) illustrating the interaction between SeO_3_^2−^ and KRAS. (D–G) Effects of SeO_3_^2−^ on GTP binding to KRAS G13D predicted by AutoDock Vina, including binding mode alterations, binding affinity, and interaction profiling (e.g., hydrogen bonds and *π*–*π* interactions). (H) Assessment of the GTP hydrolysis activity of KRAS G13D in the presence of SeNPs. (I–N) Molecular dynamics (MD) simulation analyses evaluating the conformational dynamics of KRAS upon SeO_3_^2−^ binding. (I) Backbone RMSD of KRAS in complex with SeO_3_^2−^. (J) Ligand RMSD of SeO_3_^2−^ during the simulation. (K) Root mean square fluctuation (RMSF) of individual amino acid residues. (L) Covariance matrix representing residue motion correlations. (M,N) Two‐dimensional Gibbs free energy landscapes derived from MD trajectories. (O) Thermal stability analysis of KRAS by CETSA: HCT116 cells were treated with 5 µM SeNPs or saline for 48 h, heated at indicated temperatures (37°C–67°C) for 3 min, and soluble KRAS G13D was detected by Western blot. (P) Western blot analysis of ERK1/2, p‐ERK1/2, Akt, and p‐Akt protein expression and phosphorylation levels in HCT15 and HCT116 cells treated with SeNPs (5 µM, 48 h). GAPDH was used as a loading control.

To define the molecular basis of SeNPs‐mediated inhibition of KRAS G13D‐mutant CRC, we first investigated its effect on KRAS G13D protein expression. We observed that SeNPs treatment did not alter total KRAS G13D protein levels, irrespective of the dose or duration, indicating that its mechanism of action is not at the level of protein expression but rather through direct modulation of KRAS function (Figure 4A,B). SeNPs are metabolized to SeO_3_^2−^, a phosphate analog. We hypothesized that SeO_3_^2−^ mimics phosphate by binding to the KRAS P‐loop. Molecular docking simulations strongly supported this hypothesis, revealing that SeO_3_^2−^ forms five polar contacts within 3.5 Å of the P‐loop, anchored by a Lys16 salt bridge and backbone or sidechain hydrogen bonds to Asp13, Val14, Gly15, and Ser17 (Figure 4C and Figure S10A,B). This engagement was predicted to competitively occlude GTP binding, as evidenced by a drastic reduction in KRAS–GTP hydrogen bonds (from 11 to 2), the complete loss of *π*–*π* stacking, and a 215‐fold decrease in binding affinity (Figure 4D–G). SeO_3_^2−^ also moderately weakened GDP binding (approximately 23‐fold affinity reduction); however, it was less effective, and the key *π*–*π* interactions were still preserved (Figure S10C–G), suggesting a preferential antagonism of the active GTP‐bound state. In vitro GTP hydrolysis assays functionally validated this model, demonstrating that SeO_3_^2−^ significantly diminishes the catalytic efficiency of KRAS G13D (Figure 4H). This dual interference with nucleotide binding and hydrolysis highlights SeO_3_^2−^ as a direct inhibitor of the KRAS functional cycle.

To elucidate structural dynamics underlying this inhibition, we performed a 100‐ns molecular dynamics simulation. The trajectory revealed that the KRAS G13D‐SeO_3_^2−^ complex underwent an initial conformational rearrangement before stabilizing after approximately 70 ns, as indicated by convergence of backbone Root Mean Square Deviation (RMSD) values (Figure 4I). Ligand RMSD analysis further showed that SeO_3_^2−^, following an initial exploratory phase, adopted a stable binding pose within the pocket. Notably, no ligand dissociation was observed throughout the simulation (Figure 4J). This interaction induced pronounced and localized rigidification of the P‐loop, as reflected by the reduced Root Mean Square Fluctuation values while preserving the intrinsic flexibility of the distal Switch II region (Figure 4K,L). Differential stabilization is mechanistically significant, as it enables effective inhibition at the nucleotide‐binding site without perturbing the global protein architecture, which is consistent with the stable radius of gyration (Figure S10H). The free‐energy landscape further supported this stable binding mode, showing convergence into a single, deep‐energy minimum (Figure 4M,N and Figure S10I). Hydrogen bond analysis revealed dynamic formation and dissociation (0–4 bonds) throughout the simulation despite persistent ligand retention within the binding pocket (Figure S10J). Importantly, this predicted stabilization was corroborated experimentally: a cellular thermal shift assay in HCT116 cells demonstrated that SeNPs enhanced the thermal stability of KRAS G13D (Figure 4O and Figure S10K).

In line with this, we confirmed the cellular consequences of this direct molecular inhibition. Treatment of KRAS G13D‐mutant cells with SeNPs led to a marked decrease in the phosphorylation of the key downstream effectors ERK1/2 and Akt (Figure 4P and Figure S10L,M). These findings demonstrate that SeO_3_^2−^ competitively engages the P‐loop of KRAS G13D, thereby disrupting GTP binding and hydrolysis, stably occupying the nucleotide‐binding pocket, and inducing selective rigidification of the P‐loop. This targeted inhibition of mutant KRAS activation consequently suppresses the downstream ERK/Akt signaling axis.

### 2.5 Antitumor Effects of SeNPs in CRC Xenograft Models

**FIGURE 5:**
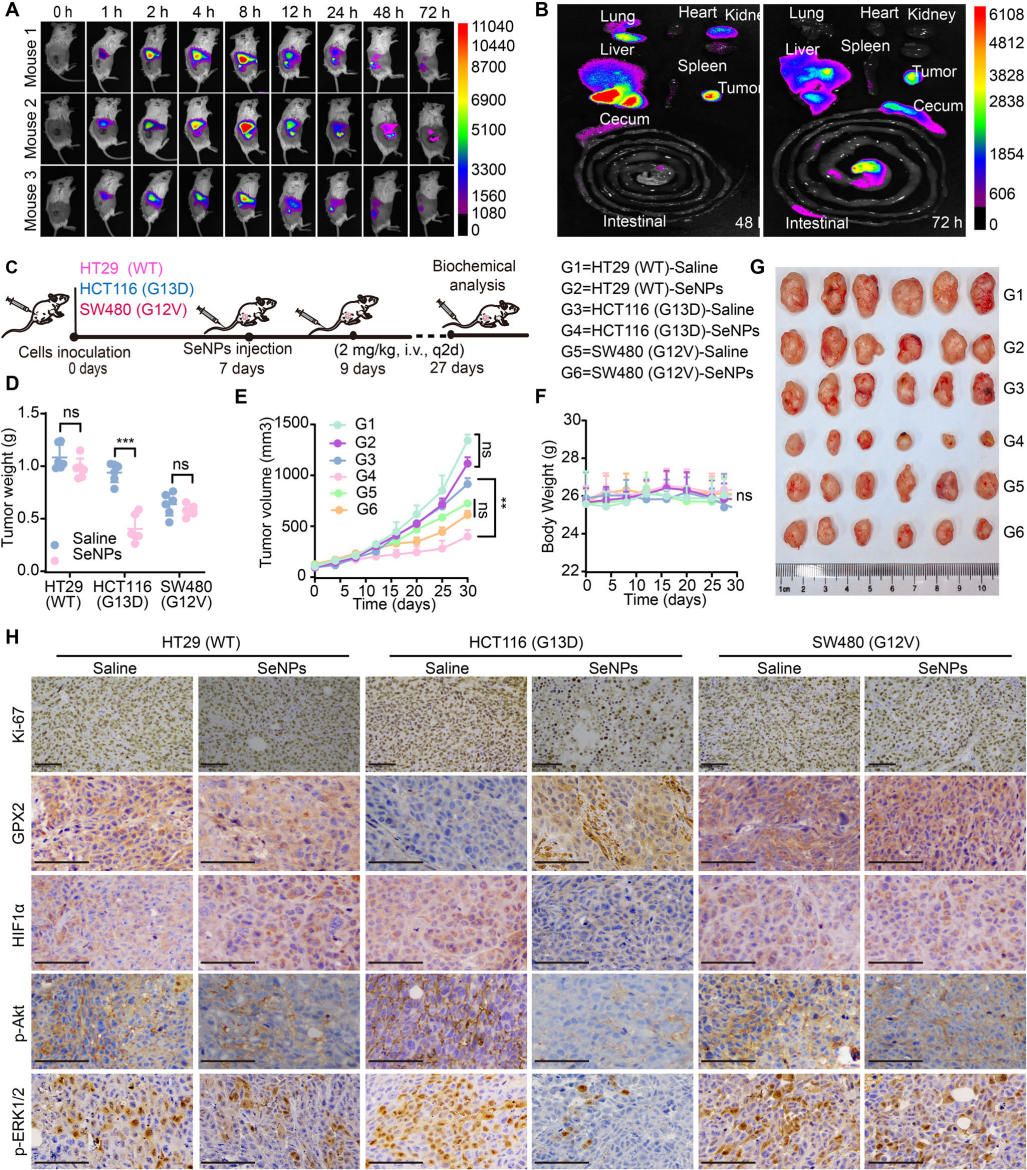
The anti‐tumor activity of SeNPs in CRC xenograft models. (A) Fluorescence imaging of tumor‐bearing mice at different times after intravenous injection of ICG‐Cy7‐SeNPs (in vivo); (B) fluorescence imaging of major organs collected at 48 h (left) and 72 h (right) (ex vivo). (C) Schematic illustration of the therapeutic effects of SeNPs (2 mg/kg, i.v., q2d) on CRC tumor‐bearing nude mice models (*n* = 6). (D) Quantitative analysis of tumor weight and (E) volume in mice with different KRAS‐mutant tumors treated with SeNPs. (F) Quantitative analysis of body weight changes in mice with different KRAS‐mutant tumors treated with SeNPs. (G) Representative tumor images of BALB/c nude mice treated with SeNPs for CRC tumor. (H) IHC staining of Ki‐67, GPX2, HIF1α, p‐Akt, p‐ERK1/2. Scale bars, 100 µm. Statistical significance was calculated using two‐way ANOVA (D–F). ns, non‐significant, ^**^ *p* < 0.01, ^***^ *p* < 0.001.

Efficient tumor delivery of SeNPs is a prerequisite for their therapeutic efficacy. Therefore, ICG‐Cy7‐SeNPs were constructed to enable real‐time tracking of SeNPs via in vivo fluorescence imaging. Following intravenous injection, fluorescence signals were initially detected in the liver and tumor sites (Figure 5A). Subsequent ex vivo imaging of major organs revealed pronounced fluorescence in the cecum (Figure 5B). These findings demonstrate the effective accumulation of SeNPs in both the tumor and cecal tissues. To assess the in vivo antitumor efficacy of SeNPs in CRC harboring the KRAS G13D mutation, xenograft models were established using CRC cell lines bearing distinct KRAS genotypes. Nude mice were subcutaneously implanted with HT29 (KRAS WT), SW480 (KRAS G12V), or HCT116 (KRAS G13D) cells to generate xenograft tumors (Figure 5C). Consistent with our in vitro observations, marked growth inhibition of KRAS G13D‐mutant tumors was noted, whereas no similar effect was observed in mice harboring KRAS WT or KRAS G12V (Figure 5D–G). Following SeNPs treatment, GPX2 expression was upregulated, whereas Ki‐67, p‐ERK1/2, p‐Akt, and HIF1α levels were significantly reduced (Figure 5H and Figure S11A–E). Moreover, staining for apoptosis markers Bax, Bad, and cleaved caspase‐3 was markedly enhanced in the KRAS G13D group, whereas no similar effects were observed in other KRAS‐mutant models (Figure S11F–I). Notably, Se supplementation effectively reversed the low GPX2 expression observed in KRAS G13D‐mutant tumors, whereas no significant changes were detected in other mutant tumors that originally exhibited high GPX2 expression. Comprehensive histopathological and serum biochemical analyses revealed no significant systemic toxicity (Figure S12A–G). In summary, these findings demonstrate that SeNPs specifically inhibit the progression of KRAS G13D‐mutant tumors via both the GPX2‐HIF1α‐VEGF pathway and the SeO_3_^2−^ ROS axis in vivo while exhibiting negligible toxicity to major organs.

### 2.6 SeNPs Inhibit Liver Metastasis of CRC in Orthotopic Xenografts Models

**FIGURE 6:**
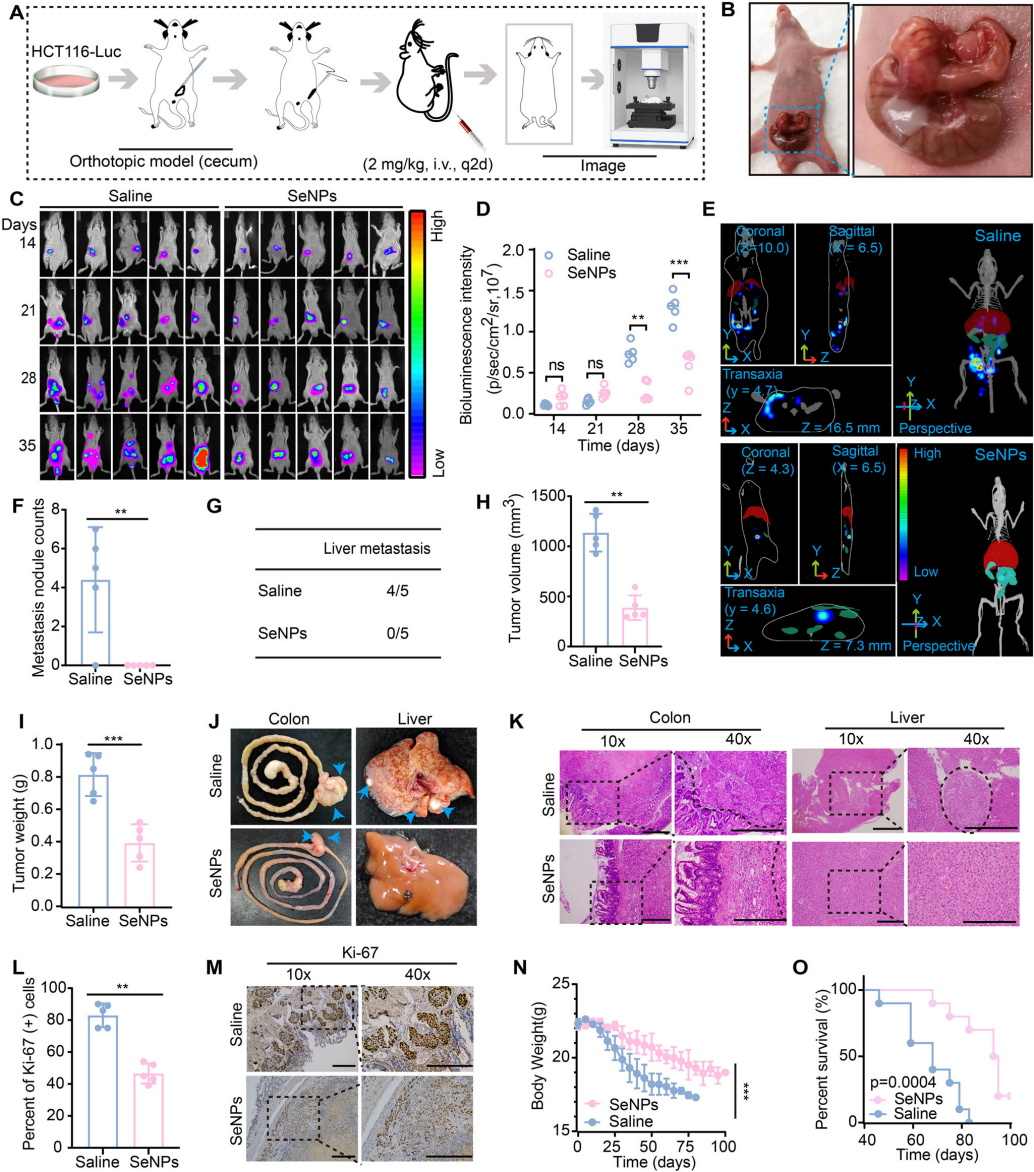
SeNPs inhibit liver metastasis of CRC in orthotopic xenografts. (A) Schematic diagram of the construction process and in vivo imaging monitoring of the orthotopic CRC model. (B) Schematic diagram of the cecum serosa implantation technique. (C) In vivo bioluminescence imaging of mice treated with saline or SeNPs (2 mg/kg, i.v., q2d) at various times and (D) quantification of bioluminescent signal intensity (*n* = 5). (E) Images of CRC liver metastasis using integrated bioluminescence imaging and 3D organ reconstruction in vivo. (F) Quantitative analysis of metastatic nodules and (G) Incidence of liver metastasis in an orthotopic CRC model. (H) Analysis of tumor volume and (I) weight. (J) Representative images of primary tumors and liver metastasis. (K) Representative images of H&E in primary tumors and liver metastases: Scale bars, 50 µm. (L) Quantified results of Ki‐67 levels and (M) Representative images of Ki‐67 staining, Scale bars, 50 µm. (N) Body weight changes in mice during treatment in different groups. (O) The survival ratios in orthotopic CRC models (*n* = 10). Statistical significance was calculated via two‐tailed Student's t‐test (F,H,I,L), the two‐way ANOVA (D,N), or the log‐rank Mantel‐Cox test (O). ns, non‐significant, ^*^ *p* < 0.05, ^**^ *p* < 0.01, and ^***^ *p* < 0.001.

Over 50% of patients with CRC develop liver metastases, and up to 90% of them ultimately succumb to metastatic disease [95, 96]. To evaluate whether the antitumor efficacy of SeNPs was retained within the orthotopic tumor microenvironment and whether they could suppress liver metastasis, we established an orthotopic xenograft model recapitulating the metastatic progression of CRC (Figure 6A) [97]. An orthotopic CRC model harboring the KRAS G13D mutation was established (Figure 6B), and treatment was initiated 2 weeks post‐injection. Tumor growth and liver metastasis were monitored weekly using a Xenogen IVIS Lumina imaging system. In the saline‐treated group, bioluminescent signals in the cecal region progressively intensified, with pronounced hepatic signals evident by day 21, indicating extensive liver metastasis. In contrast, bioluminescent signals in the SeNPs‐treated group remained constant, suggesting slower tumor progression and no detectable liver metastasis (Figure 6C,D). To further characterize metastatic dissemination in vivo, bioluminescence imaging combined with 3D organ reconstruction was performed. Multiple hepatic metastatic foci were evident in the saline group, whereas none were detected in SeNPs‐treated mice (Figure 6E). In the saline group, cecal tumors were markedly enlarged, and four of five mice developed liver metastases, with up to eight hepatic lesions per mouse. In contrast, SeNPs‐treated mice displayed no detectable metastases and also exhibited markedly reduced primary tumor volumes (Figure 6F–J). Histological analysis revealed that tumors in the saline group penetrated the muscularis and invaded the mucosal layer, whereas those in the SeNPs‐treated group remained encapsulated without breaching the muscular layer. Hematoxylin and eosin staining of liver sections revealed distinct metastatic foci in the saline group, whereas no such lesions were detected in the SeNPs‐treated group (Figure 6K). Ki‐67 expression was strongly positive in the saline group, particularly in tumor cells invading the mucosal layer, whereas only weak positivity was observed in the SeNPs‐treated group (Figure 6L,M). Body weight progressively declined in saline‐treated mice, particularly at advanced tumor stages, whereas it remained stable in the SeNPs‐treated group, indicating improved systemic conditions and reduced cachexia (Figure 6N). Survival analysis revealed a median survival time of 68 days in the saline group and 92.5 days in the SeNPs‐treated group, indicating a significant survival benefit (Figure 6O). In summary, SeNPs effectively inhibited liver metastasis of KRAS G13D‐mutant CRC, attenuated malignant progression, and markedly prolonged survival.

### 2.7 SeNPs Inhibited Tumors in PDX Models With the KRAS G13D Mutation

**FIGURE 7:**
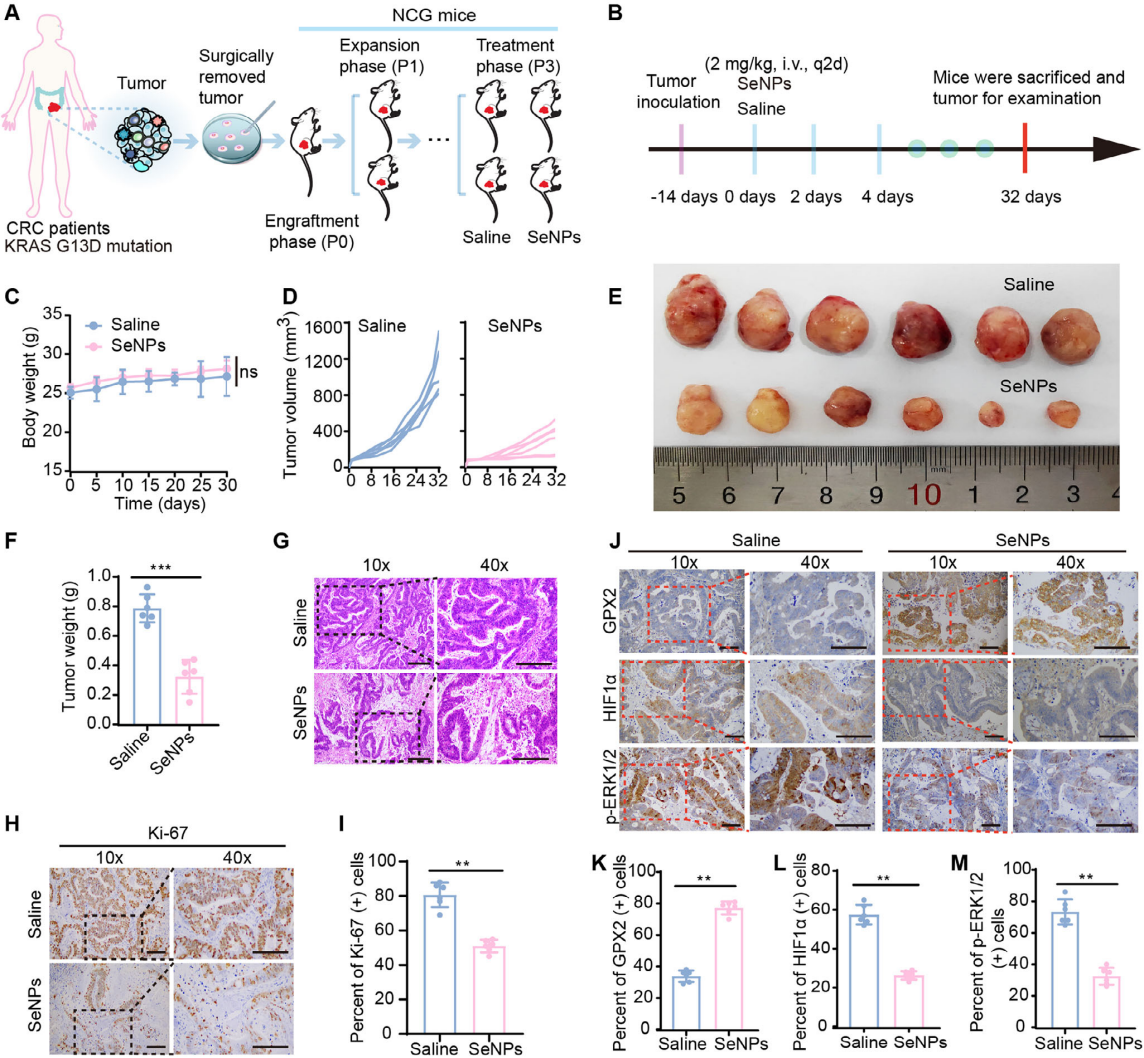
The anttumor activity of SeNPs in KRAS G13D mutant‐ CRCPDX models. (A) Schematic diagram of the establishment of KRAS G13D‐mutant PDX model. (B) Schematic of the SeNPs (2 mg/kg, i.v., q2d) therapeutic regimen in KRAS G13D‐mutant PDX mice (*n* = 5). (C) Body weight changes of PDX mice during treatment. (*n* = 5) (D) Tumor growth curves of PDX models in untreated control and SeNPs‐treated groups. (*n* = 5) (E) Representative images of tumors from KRAS G13D PDX models. (F) Analysis of tumor weight. (*n* = 5) (G) H&E staining of tumor tissues, Scale bars, 100 µm. (H,I) IHC staining (H) and quantified results (I) of Ki‑67 levels. Scale bar: 100 µm. (J–M) IHC staining and quantification of GPX2 (K), HIF1α (L), and p‐ERK1/2 (M) in control and SeNPs‐treated PDX tumors. Scale bars, 100 µm. Statistical significance was calculated via the two‐way ANOVA (C) or two‐tailed Student's t‐test (F,I,K,L,M). ns, non‐significant, ^**^ *p* < 0.01, and ^***^ *p* < 0.001.

PDX models preserve tumor heterogeneity and key oncogenic mutations, thus recapitulating tumor growth and therapeutic response more accurately [98]. The antitumor efficacy of SeNPs in KRAS G13D‐mutant CRC PDX models is expected to facilitate clinical translation (Figure 7A). Surgically resected CRC specimens harboring the KRAS G13D mutation were implanted into NCG mice to establish PDX models. The tumors were serially passaged until the third generation for ex vivo evaluation. The passaged tumor tissues showed no evidence of lymphoma transformation and maintained the histopathological features of the original tumors (Figure S13A). Sequencing analysis confirmed a missense mutation from Gly (GGT) to Asp (GAT) at codon 13 of KRAS in the PDX tumor tissues (Figure S13B). These results indicated the successful establishment of a CRC‐PDX model harboring the KRAS G13D mutation. PDX mice were divided into two groups and treated with either saline or SeNPs (Figure 7B). SeNPs treatment markedly suppressed tumor growth in the PDX model (Figure 7C–F). In the SeNPs treatment group, histological analyses revealed extensive tumor necrosis (Figure 7G); a marked reduction in proliferative activity (Ki‐67) (Figure 7H,I); decreased levels of HIF1α, p‐Akt, and p‐ERK1/2, and increased expression of GPX2 and apoptosis‐related proteins, including Bax, Bad, and cleaved caspase‐3 (Figure 7J–M and Figure S14A–E). Collectively, these findings further confirmed that SeNPs suppressed tumor progression in the PDX model through the GPX2‐HIF1α‐VEGF signaling pathway and SeO_3_^2−^–ROS axis.

## 3 Discussion

In this study, we report, for the first time, that GPX2 expression is downregulated in KRAS G13D‐mutant CRC and is associated with poor outcomes. Notably, tumor tissues harboring the KRAS G13D mutation exhibited a Se‐deficient state. To address this, we designed and synthesized SeNPs, which demonstrated potent antitumor efficacy against KRAS G13D‐mutant CRC both in vitro and in vivo. Importantly, SeNPs completely suppressed liver metastasis in the orthotopic xenograft model and achieved tumor inhibition rates of 70% and 61% in the CDX and PDX models, respectively. Mechanistically, SeNPs upregulated GPX2 expression through ΔNp63 signaling pathway and seleno‐amino acids, thereby inhibiting tumor progression through the GPX2‐HIF1α‐VEGF signaling axis. The metabolite SeO_3_^2−^ bound the P‐loop region (residues 13–17) of the KRAS G13D protein, subsequently suppressing the downstream ERK and Akt signaling. Concurrently, SeO_3_^2−^ induced cell death through the SeO_3_^2−^–ROS axis.

KRAS G13D‐mutant CRC is an especially challenging subtype, with no currently approved targeted therapies. Unlike KRAS G12C, for which covalent inhibitors such as sotorasib (AMG 510) and adagrasib (MRTX849) have shown clinical utility [99], KRAS G13D lacks druggable features. It is associated with a more aggressive clinical phenotype, including shorter median overall survival (27.7 months vs. 38.8 months) and a higher incidence of synchronous liver metastasis at diagnosis (92.1% vs. 78.2%, *p* = 0.025) [30, 31, 32]. Structural rigidity, high intrinsic activation, and increased RAF binding affinity contribute to the “undruggable” nature of KRAS G13D [24, 25, 26]. Although emerging pan‐KRAS(OFF) inhibitors, such as BI‐2865, and RAS(ON) multi‐selective inhibitors, such as daraxonrasib, have extended direct pharmacological coverage toward G13D [37, 38, 39], their translation to KRAS G13D‐mutant CRC remains constrained, as parallel inhibition of WT RAS narrows the therapeutic window. Therefore, clinical efficacy in this subtype has not yet been established. To the best of our knowledge, our study is the first to propose using SeNPs as a novel strategy to overcome this limitation.

GPX2 is a gastrointestinal‐enriched antioxidant enzyme that plays a crucial role in maintaining epithelial homeostasis and suppressing CRC progression [40, 45]. However, its function in KRAS‐mutant CRC remains poorly understood. Analysis of 306 CRC clinical specimens with various KRAS mutation subtypes revealed reduced GPX2 expression in KRAS G13D‐mutant tumors, which was associated with an advanced clinical stage and poor overall prognosis. Loss of GPX2 in this subtype disrupts ROS homeostasis and inadvertently activates pro‐metastatic pathways, potentially contributing to the observed aggressiveness and poor outcomes. Our findings suggest that SeNPs‐mediated upregulation of GPX2 suppresses the HIF1α–VEGFA axis, thereby modulating angiogenesis within the tumor microenvironment. Decreased VEGFA expression results in reduced neovascularization, thereby impairing tumor perfusion and inducing hypoxia, which in turn suppresses tumor growth. However, hypoxia may also reactivate HIF1α and VEGFA expression, thereby promoting aberrant angiogenesis and perpetuating a vicious cycle of dysfunctional vasculature and poor perfusion. Similar outcomes have also been observed in patients with breast cancer [100, 101, 102].

In this study, we demonstrate that SeO_3_^2−^ an active metabolic product of SeNPs, can selectively target and inhibit the KRAS G13D‐ mutant protein. Structurally, SeO_3_^2^^−^ exhibits a negative charge distribution and geometric configuration similar to those of PO_4_^3^^−^. The G13D mutation alters the electrostatic potential of the phosphate‐binding pocket in KRAS, which may inadvertently enhance its affinity for SeO_3_^2−^ [17, 20]. Molecular dynamics simulations further revealed that SeO_3_^2−^ forms stable, noncovalent, and dynamically maintained interactions within the P‐loop region of KRAS G13D, competitively interfering with the GTP‐binding interface. Notably, this binding mode appears to be highly selective; it effectively disrupts GTP association while still permitting GDP exchange under certain conditions, thereby enabling sustained functional inhibition of the mutant protein. Although SeO_3_^2−^ possesses therapeutic potential, high doses are associated with nonspecific oxidative damage and cytotoxicity. In contrast, SeNPs exhibit enhanced tumor selectivity and reduced systemic toxicity, with advantages primarily arising from two aspects: (1) controlled metabolic conversion, SeNPs are selectively metabolized within the reductive tumor microenvironment, leading to the sustained release of cytotoxic SeO_3_^2−^ and enabling in situ drug generation [103, 104, 105]; and (2) optimized cellular uptake, the SeNPs synthesized in this study carry a weakly positive surface charge (zeta potential of +1.5 mV), facilitating interactions with negatively charged phospholipids (e.g., phosphatidylserine) exposed on tumor cell membranes, as well as with protonated amine groups (‐NH_3_^+^), thereby promoting efficient cellular internalization [106].

SeNPs undergo distinct metabolic fates depending on the cellular redox context. Under oxidative conditions, they are predominantly converted into cytotoxic SeO_3_^2−^, whereas in normal cells, they are preferentially metabolized into selenoamino acids that support selenoprotein biosynthesis and regulation [107]. Our data support a biphasic model, in which the intracellular effects of SeNPs are both time‐ and concentration‐dependent. Following cellular uptake, SeNPs‐derived SeO_3_^2−^ rapidly induced ROS generation at early time points, thereby initiating mitochondrial apoptosis. Beginning at approximately 4 h, GPX2 synthesis was markedly upregulated, after which GPX2‐mediated ROS scavenging became dominant, leading to a reduction in ROS levels and concomitant suppression of HIF1α. Notably, GPX2 induction occurred after the initiation of apoptosis, indicating that its antioxidant activity does not interfere with early apoptotic signaling events. In addition, SeNPs exhibited a concentration‐dependent effect: at lower concentrations, GPX2 upregulation was predominant, whereas at higher concentrations, GPX2 expression was suppressed, resulting in sustained ROS accumulation.

Nevertheless, this study has certain limitations. The clinical data were derived from a single‐center retrospective cohort with a relatively small sample size, necessitating validation in larger multicenter prospective studies.

## 4 Conclusion

Our retrospective clinical analysis revealed that the selenoprotein GPX2 is markedly underexpressed in KRAS G13D‑mutant CRC and is associated with poor patient prognosis. Mechanistically, we successfully engineered SeNPs that restore GPX2 expression and suppress tumor metastasis via the GPX2–HIF1α–VEGF axis. In parallel, the in vivo metabolism of SeNPs produces selenoamino acids and SeO_3_^2−^. The latter SeO_3_^2^^−^ forms hydrogen bonds with residues 13–17 within the P‐loop of the KRAS G13D‐ mutant protein, thereby directly inhibiting downstream ERK and Akt signaling. These collective effects disrupt oncogenic KRAS signaling, suppress oxidative phosphorylation, and inhibit tumor progression. The SeNPs exhibited potent antitumor efficacy in vitro, as well as in subcutaneous xenografts, orthotopic liver metastasis, and PDX models harboring the KRAS G13D mutation, achieving favorable therapeutic outcomes. Notably, the therapeutic effect of SeNPs was selective for KRAS G13D‐mutant CRC over other KRAS variants. This mutation‐specific efficacy distinguishes SeNPs from broad‐spectrum KRAS inhibitors and supports a precision‐medicine approach for CRC patients harbouring this specific mutation. Collectively, our findings identify GPX2 as a promising therapeutic target and establish SeNPs as a translational nanomedicine for KRAS G13D‐mutant CRC, providing mechanistic insights into Se‐based nanotherapeutics and a foundation for future clinical translation.

## 5 Experimental Section

### 5.1 Ethics Approval and Consent to Participate

Patients diagnosed with CRC from The First Affiliated Hospital of Sun Yat‐sen University (FAH‐SYS, Guangzhou, China, 2024476), who received therapy from April 2015 to April 2021, were retrospectively enrolled. The inclusion criteria were as follows: (1) a pathological confirmation of CRC; (2) Detection of the KRAS mutation was performed. Exclusion criteria involved: (1) Mutations in the KRAS gene and any other gene; (2) More than one KRAS mutation site. A total of 306 eligible patients were included in the study. Clinical and pathological data, follow‐up information, and KRAS mutation status were retrospectively collected from the medical records. All procedures involving human participants were approved by the Ethics Committee of the First Affiliated Hospital of Sun Yat‐sen University (FAH‐SYSU). The study followed the ethical principles of the Declaration of Helsinki. Written informed consent was obtained from all participants. This study was reported in accordance with the STROBE guidelines.

All animal procedures were approved by the Animal Care and Use Committee of South China University of Technology (No. 2018018) and were conducted in accordance with the guidelines of the Institutional Animal Care and Use Committee, adhering to all relevant ethical regulations.

### 5.2 Cell Culture

Human CRC cell lines HT‐29 (CVCL_A8EZ), SW1116 (CVCL_0544), SW620 (CVCL_0547), HCT116 (CVCL_0291), HCT15 (CVCL_0292), and SW948 (CVCL_0632), along with the normal colon cell line FHC (CVCL_3688), were procured from ATCC (Manassas, Virginia, USA). STR analysis confirmed the identity of all cell lines. These cell lines were cultured in the medium recommended by the supplier (Gibco, CA, USA), enriched with 10% FBS (Gibco, CA, USA) and 1× penicillin‐streptomycin (Thermo Fisher Scientific, 15070063), and maintained in a 37°C incubator with 5% CO2. CRC tissues and adjacent normal mucosal tissues were sourced from the First Affiliated Hospital of Sun Yat‐sen University, with the study receiving approval from the Human Research Ethics Committee of the same institution.

### 5.3 Animal Model

All mice were acquired from GemPharmatech (Nanjing, China).

In the xenograft model, 100 µL of a cell suspension (HT29 (CVCL_A8EZ), HCT116 (CVCL_0291), SW480 (CVCL_0546), 2.0 × 10^7^ cells/mL in PBS with 20% Matrigel) was subcutaneously inoculated into the right flank of nude mice. Upon reaching a tumor volume of 80–100 mm^3^, mice with varying mutations were randomly assigned to two treatment groups: saline and SeNPs, administered intravenously every two days. Tumor dimensions (length, L; width, W) were regularly measured, with volume (V) calculated as V = L × W^2^ / 2. Tumors were imaged before surgical removal, followed by dehydration, fixation, paraffin embedding, and sectioning. Stained with hematoxylin‐eosin or IHC, samples were examined microscopically.

In the CRC orthotopic mouse model, 2 × 10^6^ cells were injected into the cecum of each nude mouse. After 14 days, mice were grouped based on bioluminescence signals, with treatments of either saline or nano‐selenium particles. Tumor progression was monitored using the IVIS Lumina Imaging System (PerkinElmer, SCR_025239), and fluorescence signals were quantified using Living Image 4.7.4. Upon the death of any mouse, all mice were euthanized, and intestinal and liver tissues were collected for analysis.

In the colorectal PDX model, fresh clinical rectal adenocarcinoma specimens (KRAS G13D mutation, microsatellite‐stable) from the First Affiliated Hospital of Sun Yat‐sen University were implanted subcutaneously into NCG mice in 3 × 3 × 3 mm fragments (2–3 per mouse). After three transplantation generations, sequencing confirmed the KRAS G13D mutation. Subsequently, 2 × 2 mm tissue pieces were transplanted into NCG mice (*n* = 6 per group). Following two weeks, mice were randomized into saline and nano‐selenium treatment groups. At the experiment's conclusion, tumor tissues were collected for histological and immunohistochemical analysis.

### 5.4 Characterization of Selenium Nanoparticles

High‐resolution TEM (HR‐TEM, JEOL 2100F SCR_020155) and TEM (Hitachi H‐7650, 100 kV, SCR_027486) were utilized for observation of the morphology and elemental distribution of the synthesized SeNPs. The structure of these nanoparticles was analyzed using x‐ray photoelectron spectroscopy (XPS, Thermoesscalab 250Xi). Particle size, zeta potential, and stability assessments were conducted with a Zetasizer Nano ZS particle analyzer (Malvern Instruments Ltd., UK). Energy‐dispersive x‐ray spectroscopy (EDS) mapping and infrared spectroscopy facilitated the determination of the composition of the SeNPs. The selenium (Se) content of the nanoparticles, after digestion with a 3:1 volume ratio of HCl and HNO_3_, was quantified using inductively coupled plasma mass spectrometry (ICP‐MS).

### 5.5 Distribution of Selenium Nanoparticles in Vivo

Indocyanine green (ICG) served as a fluorescent marker for labeling SeNPs. The biodistribution of these nanoparticles in HCT116 (CVCL_0291) xenografted nude mice was determined using real‐time imaging [108]. Dynamic imaging of the near‐infrared fluorescence signals from ICG in the mice was performed at various time intervals: 0 min, 1, 2, 4, 8, 12, 24, 48, and 72 h after injection. Upon reaching 48 and 72 h, the mice were euthanized for ex vivo imaging of the primary organs and tumors.

### 5.6 Statistical Analysis

Data were processed and analyzed using SPSS version 20.0 (IBM SPSS Statistics, Chicago, IL, USA), and graphs were generated using GraphPad Prism version 8.3 (GraphPad Software, San Diego, CA, USA). Before statistical analysis, raw experimental data were preprocessed and normalized as appropriate. For quantitative real‐time PCR (qPCR), gene expression levels were normalized to internal reference genes and calculated using the 2^‐ΔΔCt method. For Western blot analysis, protein band intensities were quantified by densitometry and normalized to the corresponding loading controls. For drug sensitivity assays, IC_50_ values were log_10_‐transformed before statistical analysis. Data are presented as mean ± standard deviation (SD) unless otherwise indicated, and the sample size (n) for each experiment was provided in the corresponding figure legends. Data distribution and homogeneity of variance were evaluated before applying parametric tests. Continuous variables were analyzed using two‐tailed Student's *t*‐test for comparisons between two groups or one‐way/two‐way analysis of variance (ANOVA) for comparisons among multiple groups, followed by appropriate post hoc multiple‐comparison tests where applicable. Immunohistochemical (IHC) scores were analyzed using the Mann–Whitney U test for comparisons between two groups. Associations between GPX2 expression and clinicopathological characteristics were assessed using the Pearson chi‐square (χ^2^) test. Survival differences were evaluated using the log‐rank (Mantel–Cox) test. All statistical tests were two‐sided, and *p* < 0.05 was considered statistically significant. Significance levels are indicated as follows: ns, not significant; ^*^ *p* < 0.05; ^**^ *p* < 0.01; ^***^ *p* < 0.001.

## Author Contributions

Conceptualization: W. L. H., T. F. C., X. T. L.; Methodology and Experiments: X. T. L., W. C., Y. Z. C., S. Y. S., S. Q. H., D. J., Y. T. H.; Investigation: X. T. L., Y. M., Q. H. L.; Writing – original draft: X. T. L., W. C., Y. Z. C.; Writing – review & Editing: W. L. H., T. F. C., C. W., X. T. L., L. M.; Funding acquisition: W. L. H., T. F. C.; Resources: W. L. H., T. F. C., C. W., X. T. L., Y. Z. C.; Supervision: W. L. H., T. F. C.; Project management: W. L. H., T. F. C.; Visualization: X. T. L., C. W.

## Funding

This work was supported by the National Natural Science Foundation of China (92359302, 82472087), Natural Science Foundation of Fujian Province (2024J011004), Fujian Provincial Health and Medical High‐Level Talent Team (XM050005), Natural Science Foundation for Outstanding Youth Team Project of Guangdong Province (2024B1515040030), Key‐Area Research and Development Program of Guangdong Province (2023B1111020005).

## Conflicts of Interest

The authors declare no conflicts of interest.

## Supporting information




**Supporting File 1**: advs77004‐sup‐0001‐SuppMat.docx.

**Supporting File 2**: advs77004‐sup‐0002‐DataSet.docx.

## Data Availability

Research data are not shared.
