# Supplementary material for: Selenium Nanoparticles Selectively Target KRAS G13D to Inhibit Colorectal Cancer
Source: Adv Sci (Weinh). 2026 Aug 18:e23795. Online ahead of print. doi: 10.1002/advs.202523795 (PMC13483143; doi:10.1002/advs.202523795)
Supplement: Supplementary file 1 [file ADVS-9999-e23795-s001.docx]

Figure 1I

Figure 3D

Figure 3F

Figure 3G

Figure 3J

Figure 3K

Figure 3L

Figure 3M

Figure 4A

Figure 4B

Figure 4O

Figure 4P

Figure S9G
