# Supplementary material for: Selenium Nanoparticles Selectively Target KRAS G13D to Inhibit Colorectal Cancer
Source: Adv Sci (Weinh). 2026 Aug 18:e23795. Online ahead of print. doi: 10.1002/advs.202523795 (PMC13483143; doi:10.1002/advs.202523795)
Supplement: Supplementary file 2 [file ADVS-9999-e23795-s002.docx]

Supporting Information

Title: Selenium Nanoparticles Selectively Target KRAS G13D to Inhibit Colorectal Cancer

*Xiaoting Liu, Chong Wu, Shiyao Song, Li Ma, Shaoqing Huang, Yuting He, Qinghai Li,* [*Yanzhou Chang*](https://pubmed.ncbi.nlm.nih.gov/?term=Chang+Y&cauthor_id=37204161)*, Dan Jiang, Yong Mei, Junchao Cai, Zhenshuang Du,^*^* [*Tianfeng Chen*](https://pubmed.ncbi.nlm.nih.gov/?term=Chen+T&cauthor_id=37204161)*,^*^ and Weiling He^,*^*

*Xiaoting Liu, Yuting He, Zhenshuang Du and Weiling He*

Department of Gastrointestinal Surgery, Xiang'an Hospital of Xiamen University, School of Medicine, Xiamen University, Xiamen, Fujian, 361000, China

Email: zsdu@xah.xmu.edu.cn (Z.-S. Du), [wlhe@xah.xmu.edu.cn](mailto:wlhe@xah.xmu.edu.cn) (W.-L. He).

*Xiaoting Liu, Chong Wu, Shaoqing Huang, Qinghai Li, Yong Mei* and *Weiling He*

Department of Gastrointestinal Surgery, The First Affiliated Hospital, Sun Yat-sen University; Guangzhou, Guangdong 510080, China

*Li Ma*, [*Yanzhou Chang*](https://pubmed.ncbi.nlm.nih.gov/?term=Chang+Y&cauthor_id=37204161), *Dan Jiang*, *and* [*Tianfeng Chen*](https://pubmed.ncbi.nlm.nih.gov/?term=Chen+T&cauthor_id=37204161)

Department of Chemistry, College of Chemistry and Materials Science, Jinan University, Guangzhou, 510632, China.

Email: [tchentf@jnu.edu.cn](mailto:tchentf@jnu.edu.cn) (T.-F. Chen)

*Shiyao Song*

Department of Pediatrics, The First Affiliated Hospital, Sun Yat-sen University, Guangzhou, Guangdong, 510080, China

*Junchao Cai*

Department of Immunology, Zhongshan School of Medicine, Sun Yat-sen University, Guangzhou, Guangdong, 510080, China.

*Xiaoting Liu, Chong Wu, and Shiyao Song* contributed equally to this work

Supplementary Figures

**Supplementary Figure 1. Association between GPX2 expression levels and survival outcomes in CRC patients.**
Kaplan-Meier survival curves comparing patients with high versus low GPX2 expression for: A) Progression-free survival (PFS). B) Recurrence-free survival (RFS). C) Overall survival (OS).

**Supplementary Figure 2. Expression patterns of GPX2 in CRC**.

A) Expression levels of GPX2 in tumor tissues versus normal intestinal epithelium from the TCGA database. B) GPX2 expression in metastatic versus primary CRC samples from the GEO dataset (GSE35834). C) GPX2 expression scores across different clinical stages of CRC. Statistical significance was analyzed by Unpaired two-tailed Student's t-test (C), * *p* < 0.05, ** *p* < 0.01.

**Supplementary Figure 3. Association between GPX2 expression levels and survival outcomes in CRC patients.**

A and B) Kaplan-Meier analysis of (A) PFS and (B) OS according to GPX2 expression (high *vs*. low). The log-rank Mantel-Cox test (A, B).

**Supplementary Figure 4.** qPCR analyses of GPX2 expression in normal intestinal epithelial cells, KRAS wild-type cells, and CRC cell lines harboring different KRAS mutations.

**Supplementary Figure 5. Structural characterization and physical property studies of SeNPs.**

A) Particle size of selenium nanoparticles. B) Zeta potential of selenium nanoparticles. C) FTIR spectrum of selenium nanoparticle. D) Full XPS spectrum of selenium nanoparticles. E and F) C 1s and O 1s high-resolution XPS spectrum of selenium nanoparticles. G) Stability of SeNPs under different culture conditions (*n* = 3).

**Supplementary Figure 6. SeNPs significantly inhibits the proliferation, migration, and invasion of KRAS-G13D mutant CRC cells**.

A) Representative images and quantitative analysis of colony formation assays in KRAS-mutant CRC cells treated with 5 μM SeNPs for 48 h (*n* = 3). B) The representative images and quantitative analysis of the Matrigel invasion assay of CRC cells with KRAS mutation treated with 5 μM SeNPs for 48 h (*n* = 3). C to E) Representative images and quantitative analysis of wound-healing assay of CRC cells with KRAS mutation treated with 5 μM SeNPs for 48 h (*n* = 3). Statistical significance was analyzed by Unpaired two-tailed Student's t-test (A-E), ** *p* < 0.01, ****p* < 0.001.

**Supplementary Figure 7. Biotransformation and metabolic characteristics of SeNPs.**

A) Time-dependent uptake of SeNPs (5 μM) in different CRC cell lines (*n* = 3). B) Quantitative analysis of intracellular Se species at different time points determined by HPLC–ICP-MS. Data are presented as mean ± SD (*n* = 3). C)Time-course analysis of GPX2 mRNA expression in HCT116 cells treated with 5 μM SeNPs. Data are presented as mean ± SD (*n* = 3). Statistical significance was calculated using two-way ANOVA (B, C), or one-way ANOVA (A). **p* < 0.05, ***p* < 0.01, ****p* < 0.001.

**Supplementary Figure 8.** A and B) Gene expression correlation analysis: (A) GPX2 with

VEGF, and (B) HIF1α with VEGFC.

**Supplementary Figure 9. SeNPs induce mitochondrial dysfunction.**

A) GSEA pathway enrichment analysis of the top 10 downregulated genes following SeNPs (5 μM, 48 h) treatment. B) GSEA enrichment analysis of the electron transport chain (OXPHOS) pathway. C) Heatmap of mitochondrial-related gene expression in SeNPs-treated and control groups in HCT116 cells (5 μM, 48 h). D) Representative fluorescence images showing mitochondrial morphology labeled with MitoTracker in HCT116 cells (5 μM, 48 h, *n* = 3). Scale bar: 50 μm. E) Representative TEM images showing cellular ultrastructure, along with quantitative analysis of mitochondrial number in HCT116 cells (5 μM, 48 h), yellow arrows indicate damaged mitochondria. Scale bar: 500 nm. F) Analysis of cell apoptosis by Annexin V/PI double staining combined with flow cytometry in HCT116 cells (5 μM, 48 h). G) Western blot analysis of Bad, Bax, Caspase-3, and cleaved Caspase-3 protein levels under GPX2 overexpression or SeNPs treatment conditions in HCT116 cells (5 μM, 48 h). GAPDH was used as a loading control. H) ATP level measurement after in HCT116 cell line following treatment with SeNPs at various concentrations. (n = 3). Statistical significance was calculated by Unpaired two-tailed Student's t-test (E) or one-way ANOVA (H), *** *p* < 0.001.

**Supplementary Figure 10. SeNPs-derived selenite competitively disrupts GTP binding and hydrolysis of KRAS G13D.**

A) Molecular docking using AutoDock Vina 1.2.7 reveals the binding modes of KRAS G13D with GTP, GDP, and SeO₃²⁻. B) Molecular docking interaction results of SeO₃²⁻ with key residues in the P-loop region of the KRAS G13D mutant. C–F) Effects of SeO₃²⁻ on GDP binding to KRAS G13D predicted by AutoDock, including binding mode alterations, binding affinity, and interaction profiling (e.g., hydrogen bonds and π–π interactions). G) Effects of SeO₃²⁻ binding to KRAS WT predicted by AutoDock. H) Radius of gyration (Rg) analysis of the KRAS G13D–selenite complex during molecular dynamics simulations. The overall Rg (red) and its vector components (Rg/X, Rg/Y, and Rg/Z) are plotted as a function of simulation time to assess global compactness and directional conformational fluctuations. I) Interaction energy between KRAS G13D and selenite (SeO₃²⁻) derived from molecular dynamics simulations. Energy values are expressed in kJ/mol. J) Number of hydrogen bonds between KRAS G13D and selenite (SeO₃²⁻) during molecular dynamics simulations. K) quantification of KRAS band intensities relative to the 37 °C band (set to 1.0). (*n* = 3). L) Relative p-Akt/Akt ratios in cells following SeNPs treatment (5 μM, 48 h) were quantified by densitometric analysis of Western blot bands. Data are presented as mean ± SD (*n* = 3). M) Relative p-ERK1/2/ERK1 ratios in cells following SeNPs treatment (5 μM, 48 h) were quantified by densitometric analysis of Western blot bands. Data are presented as mean ± SD (*n* = 3). Statistical significance was calculated via Unpaired two-tailed Student's t-test (K-M). ns, non-significant, ***p* < 0.01, ****p* < 0.001.

**Supplementary Figure 11. The antitumor activity of SeNPs.**

A to E) The quantified results of (A) Ki-67, (B) GPX2, (C) HIF1α, (D) pERK1/2, (E) p-Akt. Data are represented as means ± SD (*n* = 6). F to I) H&E, IHC staining and quantification of Bad, Bax, and cleaved caspase-3 of tumor tissues with different KRAS mutations after SeNPs treatment. Scale bars, 100 μm. (*n* = 6) Statistical significance was analyzed by Unpaired two-tailed Student's t-test, ** *p* < 0.01, ****p* < 0.001.

**Supplementary Figure 12. Biosafety evaluation of SeNPs.**

A to F) Blood biochemical parameters in tumor-bearing mice at the end of the study**,** Comparison of blood biochemical parameters in tumor-bearing mice treated with saline or SeNPs (*n* = 6): A) uric acid, B) blood urea nitrogen, C) alanine aminotransferase, D) aspartate aminotransferase, E) triglycerides, and F) serum creatine kinase. G) H&E staining of major organs showing histological changes post-treatment in mice bearing CRC cells with different KRAS mutations. Statistical significance was analyzed by Unpaired two-tailed Student's t-test, ns, non-significant.

**Supplementary Figure 13. Histological and genotypic validation of KRAS G13D-Mutant PDX model.**

A) Representative images of H&E, CK, CD45, and CD20 staining in PDX tumor tissues. Scale bars, 50 μm. B) Sanger sequencing chromatogram confirming the KRAS G13D mutation.

**Supplementary Figure 14. The anti-tumor activity of selenium nanoparticles (SeNPs) in the PDX model**.

A) The representative images of p-Akt, Bad, Bax, and cleaved caspase 3 (CC3) staining in tumor tissues from KRAS-G13D CRC-PDX mouse models treated with SeNPs. Scale bars,

50 μm. B to E) The quantified results of B) p-Akt, C) Bad, D) Bax, and E) cleaved caspase 3 levels. Data are represented as means ± SD (*n* = 6). Statistical significance was analyzed by Unpaired two-tailed Student's t-test, ns, non-significant, ***p* < 0.01 levels.

Supplementary Methods

**PDX Genetic and Immunohistochemical Validation**

In patient-derived xenograft (PDX) models established in NCG mice, tumor tissues were analyzed to confirm their fidelity to the parental tumor tissue. This analysis included H&E and IHC staining to exclude lymphoma in the PDX models, as well as sequencing of the KRAS mutation site for genetic characterization. Tumor samples from CRC-PDX xenografts were fixed in neutral formalin, embedded in paraffin, sectioned, and subjected to immunohistochemical analysis using a Leica automated immunohistochemistry system (BONO-MAX). The antibodies used included anti-human pan-cytokeratin (Cell Signaling Technology, #4545S, AB_490860), anti-human CD45 (Dako, #PR70101, AB_3675500), and anti-human CD20 (Dako, #M0755, AB_2282030). IHC results confirmed a pan-cytokeratin-positive/CD45-negative/CD20-negative phenotype, while sequencing identified a KRAS G13D mutation, consistent with the original tumor tissue from the patient. These findings confirmed the preservation of the genetic and pathological characteristics of the parental tumor.

**Immunohistochemical staining and scoring**

First, the GPX2 antibody (ab137431, Abcam, AB_3674655) was used to detect GPX2 expression in FFPE samples from CRC patients. Similarly, various antibodies were used to analyze FFPE samples from CRC-PDX xenograft tumors harboring the KRAS G13D mutation, including antibodies against Ki-67 (9449, Cell Signaling Technology, AB_2797703), cleaved caspase-3 (9661S, Cell Signaling Technology, AB_2341188), Bax (14796S, Cell Signaling Technology, AB_2716251), Bad (9268S, Cell Signaling Technology, AB_10695002), phosphorylated Akt (Ser473) (4060, Cell Signaling Technology, AB_2315049), and phosphorylated p44/42 MAPK (Erk1/2) (Thr202/Tyr204) (4370, Cell Signaling Technology, AB_2315112). After incubation with the primary antibodies, DAB+ chromogen was used for antigen detection. ImageJ software (SCR_003070) was used to quantify the staining by assessing the percentage of positively stained cells and staining intensity in high-power fields. Staining intensity was classified into four levels: 0 (no staining), 1+ (weak staining visible at high magnification), 2+ (moderate staining), and 3+ (strong staining visible even at low magnification).

**Western blotting**

Western blotting was performed by lysing cells with RIPA buffer, followed by centrifugation at 12,000 rpm for 30 minutes at 4°C. The supernatant was collected, and protein concentrations were determined using a BCA protein assay kit. The lysates were then subjected to SDS-PAGE and transferred to a PVDF membrane (Merk Millipore, IPVH00010). Primary antibodies targeting Akt (2966, Cell Signaling Technology, AB_823417), Erk1/2 (9101, Cell Signaling Technology, AB_331646), phosphorylated Akt (Ser473) (4060, Cell Signaling Technology, AB_2315049), and phosphorylated p44/42 MAPK (Erk1/2) (Thr202/Tyr204) (4370, Cell Signaling Technology, AB_2315112), GPX2, HIF1a, VEGF, GAPDH (97166, Cell Signaling Technology, AB_2756824), DeltaNp63 (ab203826, abcam, AB_2934266), and Ras (G13D Mutant) Antibody (PA5-78088, Thermo Fisher, AB_2736502) were used for immunoblotting. HRP-conjugated secondary antibodies (anti-rabbit/mouse IgG) were applied to facilitate detection.

**RNA isolation, reverse transcription, and quantitative real-time PCR (qPCR)**

Total RNA was isolated from cells using TRIzol reagent (Invitrogen, CA, USA), and cDNA was synthesized with the PrimeScript RT reagent kit (TaKaRa, Dalian, China). Quantitative PCR (qPCR) assays were performed using the SYBR Green Real-Time RT-PCR Kit (TaKaRa, Dalian, China) on an ABI7500 Real-Time PCR System (Applied Biosystems, CA, USA). For each template, Ct values were determined in triplicate, and their mean and standard deviation were computed as experimental results. Both the internal control gene and target gene Ct values were measured for each sample. The ΔCT was calculated as the average Ct of triplicates minus the average Ct of the corresponding internal control. The ΔΔCT, representing the fold change in target gene expression between experimental and control groups, was determined by comparing the ΔCT values between these groups. The 2^-ΔΔCT method was used to calculate the fold difference. All data were normalized against GAPDH expression as an internal reference. The specific primer pairs used for qPCR are detailed in Appendix Table S1.

**Cell Counting, Colony Formation, Wound Healing, and Transwell Assays**

For cell enumeration, cultured cells were dissociated with 0.25% trypsin and counted using a cell counter. For colony formation assays, 2,000 cells per well were seeded in 6-well plates, treated with the indicated drugs after cell adhesion, and incubated for 2 weeks. The cells were then fixed with 4% paraformaldehyde and stained with Giemsa for colony enumeration. For wound healing assays, cells cultured in 6-well plates were scratched to create a uniform monolayer gap, treated with the indicated drugs, and imaged at 0 and 48 h using a fluorescence microscope to assess cell migration. Serum-free medium was used throughout these assays. For Transwell assays, 10,000 cells per well were suspended in serum-free medium and seeded into the upper chambers of 8-µm-pore Transwell inserts. The lower chambers contained medium supplemented with 10% FBS as a chemoattractant, and the indicated drugs were added to the upper chambers. After incubation at 37°C for 48 h, cells that had migrated through the pores were fixed and stained with Giemsa for 15 min. The stained cells were imaged at 200× magnification in five randomly selected fields and quantified using ImageJ (NIH). For invasion assays, Matrigel (Corning, NY, USA) was added to the upper chambers of the Transwell inserts. Data from three independent experiments were analyzed using a two-tailed Student’s t-test.

**Morphological changes of mitochondria**

The Mitotracker and DAPI staining assay was utilized to monitor the morphological changes in mitochondria. HCT116 (CVCL_0291) cells were treated with SeNPs (5 μM) for various durations, then incubated with Mitotracker (100 nM, CM-H2Ros) for 1 hour, followed by a 10-minute incubation with DAPI (1 mg/mL). After incubation, excess Mitotracker, DAPI, and drugs were washed off three times with PBS. Subsequently, mitochondrial morphological changes were observed using a Zeiss LSM 880 Airyscan confocal microscope (Carl Zeiss, Oberkochen, Germany, SCR_020925) equipped with a 63x objective.

**KRAS Mutation Analyses**

DNA was extracted from formalin-fixed paraffin-embedded (FFPE) or fresh tumor tissues. KRAS mutations were then detected using a polymerase chain reaction clamping and melting curve technique, as described previously.

**Cellular uptake of selenium nanoparticles**

HCT116 (CVCL_0291) cells were cultured in 6-well plates, after which the medium was discarded and replaced with fresh medium containing SeNPs (5 μM). Following a 48-hour incubation in the selenium-rich medium, the cells were washed with PBS buffer. The harvested cells were then digested using 3 mL of concentrated nitric acid and 1 mL of hydrogen peroxide in an infrared rapid digestion system (Gerhardt) at 180 °C for 1.5 hours. The digested solution was diluted to a final volume of 10 mL with Milli-Q water, and the selenium (Se) concentration was determined using Inductively Coupled Plasma Mass Spectrometry (ICP-MS).

***In vivo* optical imaging**

Before *in vivo* imaging, mice were anesthetized with isoflurane. For bioluminescence imaging, they received an intraperitoneal injection of d-luciferin solution (150 mg/kg), applicable for in situ xenograft studies. Imaging was performed 15 minutes later using the IVIS Lumina Imaging System (PerkinElmer), with exposure times between 10 and 30 seconds. Fluorescence imaging was conducted at an excitation wavelength of 465 nm and an emission wavelength of 520 nm. Bioluminescence and fluorescence signals were quantitatively assessed on days 14, 21, 28, and 35 using Living Image 4.7.4 (Caliper Life Sciences, Alameda, USA, ImageJ software).

**Transmission electron microscopy (TEM)**

HCT15 and HCT116 cells were treated with SeNPs (5 μM) for 48 h, then fixed overnight at 4°C in 2.5% glutaraldehyde, followed by a 1-hour fixation in 1% osmium tetroxide. After dehydration in acetone, the cells were embedded in resin, sectioned with an ultramicrotome, double-stained with uranyl acetate and lead citrate, and subsequently examined under a transmission electron microscope (Japan, JEOL, 2100F, SCR_020155).,

**HPLC-ICP-MS Assay**

Selenium metabolism was analyzed using HPLC-ICP-MS. Specifically, 1.9213 g of citric acid was dissolved in 1 L of Milli-Q water to prepare a 10 mmol/L solution, which was then adjusted to pH 4.50 with ammonium hydroxide (25 wt.%). The solution was subsequently filtered through a 0.22-µm membrane to prepare the mobile phase. A 400-µL sample was manually injected for each analysis. Isocratic elution was performed at a flow rate of 0.8 or 0.6 mL/min for 1500 s to detect selenium compounds. Standards of SeCys2, SeMC, SeMet, SeO₃²⁻, and SeO₄²⁻ at concentrations of 0, 25, 50, 100, and 200 µg/L were used to quantify the selenium metabolites of Cys@SeNPs using a Thermo Scientific iCAP RQ ICP-MS coupled with an UltiMate 3000 UHPLC system. The collected data were automatically analyzed using Qtegra software, and the results were visualized using GraphPad Prism 8.3.**Cell Viability Assay**

CRC cells with different KRAS mutations were seeded into 96-well plates at a concentration of 1-2 × 10^4^ cells/mL (100 μL) and incubated for 12 hours. Subsequently, the cells were treated with various concentrations of selenium-containing drugs (3.125, 6.25, 12.5, 25, 50,100 µM) for 48 hours. Cell toxicity and IC_50_ values were then evaluated using the CCK-8 assay.

**Cell culture**

Human CRC cell lines HT-29, SW1116, SW620, HCT116, HCT15, and SW948, as well as the normal colon cell lines FHC and NCM460, were obtained from ATCC (Manassas, VA, USA). The identity of all cell lines was confirmed by short tandem repeat (STR) analysis. The cell lines were cultured in the media recommended by the supplier (Gibco, CA, USA), supplemented with 10% FBS (Gibco, CA, USA) and 1× penicillin-streptomycin (Thermo Fisher Scientific, 15070063), and maintained at 37°C in a humidified incubator with 5% CO₂. CRC tissues and adjacent normal mucosal tissues were obtained from the First Affiliated Hospital of Sun Yat-sen University. The study was approved by the Human Research Ethics Committee of the First Affiliated Hospital of Sun Yat-sen University.

**Annexin V/PI apoptosis assay**

Apoptosis was assessed using the Annexin V-APC/PI Apoptosis Detection Kit (Keygen Biotech, Nanjing, China; Cat# KGA1030-100) according to the manufacturer's instructions. HCT116 and HCT15 cells were treated with SeNPs (5 μM) or vehicle control for 48 h. Cells were harvested by trypsinization, washed twice with cold PBS, and resuspended in 1× binding buffer at approximately 1 × 10⁶ cells/mL. A 100 μL aliquot was incubated with 5 μL Annexin V-APC and 5 μL PI at room temperature for 15 min in the dark, followed by addition of 400 μL binding buffer. Samples were immediately analyzed on a Beckman Coulter CytoFLEX S flow cytometer, and data were analyzed using FlowJo. The sum of early apoptotic (Annexin V⁺/PI⁻, Q1-LR) and late apoptotic (Annexin V⁺/PI⁺, Q1-UR) cells was quantified as the total apoptosis rate. Experiments were performed in three independent biological replicates.

**Plasmid and siRNA transfection**

GPX2 overexpression was achieved by transient transfection of a pCDH-CMV-MCS-EF1-copGFP vector (System Biosciences) into which the full-length human GPX2 coding sequence had been cloned at the MCS. GPX2 knockdown was performed using a GPX2-targeting siRNA (si-GPX2), with a non-targeting scrambled siRNA as the control. Cells were seeded in 6-well plates at approximately 70% confluence one day prior to transfection. Plasmid transfection was performed with Lipofectamine 3000 (Invitrogen, cat# L3000015) following the manufacturer's protocol. siRNA transfection was performed with Lipofectamine RNAiMAX (Invitrogen, cat# 13778150) at a final siRNA concentration of 50 nM. After 48 h of transfection, cells were treated with 5 μM SeNPs for an additional 48 h before harvest for Western blot analysis.

**RNA sequencing and bioinformatics analysis**

HCT116 cells were treated with 5 μM SeNPs or vehicle control for 48 h (*n* = 3 biological replicates per group). Total RNA was extracted with TRIzol reagent (Invitrogen) following the manufacturer's protocol, and its integrity and purity verified with a NanoDrop ND-1000 spectrophotometer and an Agilent Bioanalyzer 2100. Strand-specific sequencing libraries were prepared from 1 μg of total RNA with the NEBNext Ultra RNA Library Prep Kit for Illumina (NEB, Cat. E7530L) according to the manufacturer's instructions, and sequenced on an Illumina NovaSeq 6000 platform (IGE Biotechnology, Guangzhou, China) to generate 150-bp paired-end reads.

Raw FASTQ reads were processed with fastp to remove adapters and low-quality bases. Clean reads were aligned to the human reference genome (GRCh38) with HISAT2 v2.0.5, and gene-level counts and FPKM values were quantified with RSEM. Differential expression analysis was performed with the DESeq2 R package (v1.38) using a negative binomial model with the design formula ~ condition; P-values were adjusted by the Benjamini-Hochberg method, and genes with adjusted P < 0.05 and |log₂ fold change| ≥ 1 were considered differentially expressed, identifying 890 upregulated and 640 downregulated genes. Gene Set Enrichment Analysis (GSEA) was performed with the clusterProfiler R package (v4.x) using gene sets from MSigDB, with Benjamini-Hochberg adjusted P < 0.05 considered significant.

**Molecular Dynamics Simulation (Comprehensive Version)**

Based on the small molecule–protein complex (SeO₃²⁻–KRAS G13D) obtained from molecular docking, all-atom molecular dynamics simulations were performed using the GROMACS software package. The CHARMM36m force field was employed for parameterization. The complex was placed in a cubic box with a minimum distance of 1.0 nm between the box boundaries and the molecules, and the system was solvated using the TIP3P water model. Subsequently, Na⁺/Cl⁻ ions were added to neutralize the total charge of the system.

The system was first subjected to energy minimization using the steepest descent method to eliminate unreasonable atomic contacts. This was followed by NVT (constant number of particles, volume, and temperature) equilibration and NPT (constant number of particles, pressure, and temperature) equilibration sequentially. The production MD simulation was conducted under three-dimensional periodic boundary conditions for a total duration of 100 ns with an integration time step of 2 fs. Long-range electrostatic interactions were treated using the Particle Mesh Ewald (PME) method, while short-range electrostatic and van der Waals interactions were calculated with a cutoff radius of 1.0 nm. All bonds involving hydrogen atoms were constrained using the LINCS algorithm. The system temperature was maintained at 300 K using the V-rescale thermostat, and the pressure was maintained at 1.0 bar using the Berendsen barostat. Trajectory and energy information were saved every 30 ps during the simulation.

After the simulation, trajectory files were processed using gmx trjconv to correct for periodic boundary conditions, and the protein was centered to minimize boundary artifacts. Based on the corrected trajectories, dynamic parameters including RMSD, RMSF, radius of gyration (Rg), solvent-accessible surface area (SASA), and the number of protein–ligand hydrogen bonds were calculated. Principal component analysis (PCA) was performed on the processed trajectories based on protein backbone atoms to extract the dominant conformational motion patterns of the system. A residue motion correlation heatmap was constructed through covariance matrix analysis to characterize the correlated motion patterns of residues within the protein. A three-dimensional Gibbs free energy landscape (FEL) was constructed based on the first two principal components to identify low-energy stable conformations.

**Molecular docking**

The KRAS G13D X-ray structure (PDB 4TQA, chain A) was used as the receptor. GDP and GTP were extracted from the same structure and prepared with Meeko; selenite (SeO₃²⁻) was generated from the SMILES string [O-][Se](=O)[O-] using RDKit and minimised with MMFF94. Docking was performed with AutoDock Vina using a 22 × 22 × 22 Å³ search box centred on the crystallographic GDP centroid of chain A (exhaustiveness = 16, num_modes = 9, seed = 42), and the top-ranked pose (MODE 1) of each ligand was retained for downstream analysis. To probe whether a pre-bound SeO₃²⁻ perturbs subsequent nucleotide engagement, GDP and GTP were re-docked into the SeO₃²⁻-occupied receptor (SeO₃²⁻ MODE 1 appended as a HETATM cofactor) under identical box, seed and exhaustiveness settings; ΔΔG was defined as ΔG (with SeO₃²⁻) – ΔG (without SeO₃²⁻), and predicted dissociation constants were calculated as Kd = exp (ΔG / RT) at T = 298.15 K. All Kd values are docking-derived predictions rather than biophysical measurements.

Protein-ligand interaction fingerprints for GDP and GTP were computed with ProLIF on each top-ranked pose, tabulating hydrogen-bond donors/acceptors, π-π stacking and van der Waals contacts in both the ligand-free and SeO₃²⁻-occupied receptors. Polar contacts between SeO₃²⁻ and the receptor were quantified directly from atomic coordinates in PyMOL using the cmd.distance function with a 3.5 Å cutoff, and the wider contact shell was defined as all residues with any atom within 5 Å of SeO₃²⁻

**GTPase Activity Using Phosphate Sensor Assay Kit**

The GTPase activity of KRAS proteins was measured using the Phosphate Sensor Assay Kit (Beyotime, S0192S/M), which employs a fluorescence-labeled recombinant phosphate-binding protein for ultrasensitive detection of inorganic phosphate and enzymatic activities including ATPases, GTPases, phosphatases, and phosphodiesterases. The assay was performed in black 384-well plates (Corning). In each well, 10 μL of 1× assay buffer (supplemented with MgCl₂ to a final concentration of 5 mM) was added, followed by 10 μL of either 1 μM KRAS G13D mutant protein or 1 μM KRAS wild-type protein, along with increasing concentrations of SeNPs (0.78–100 μM). The mixture was pre-incubated at 25°C for 60 minutes to allow sufficient interaction between SeNPs and KRAS proteins. Subsequently, GTP was added to initiate the hydrolysis reaction. After 60 minutes of hydrolysis, the reaction was terminated by adding EDTA to a final concentration of 20 mM, and the samples were appropriately diluted to ensure the phosphate concentration fell within the linear range of the standard curve. Then, 10 μL of the diluted reaction mixture was mixed with 10 μL of 10 μM Phosphate Sensor working solution (freshly diluted in 1× assay buffer) in a black 384-well plate. Fluorescence intensity was immediately measured using a microplate reader (e.g., BioTek Synergy H1) at an excitation wavelength of 430 nm and an emission wavelength of 450 nm. The phosphate concentration was calculated based on a standard curve generated using the phosphate standard provided with the kit.

**Cellular Thermal Shift Assay**

Cells were treated with 5 μM SeNPs for 48 h, then collected, washed three times with ice‑cold PBS, and resuspended in PBS containing protease inhibitors (without detergents). The cell suspension was aliquoted into EP tubes and placed in a heat block. The tubes were heated at designated temperatures ranging from 37 °C to 67 °C (at 3 °C intervals) for 3 min, followed immediately by cooling on ice for 3 min. After cooling, an equal volume of mild lysis buffer was added to each tube, and the cells were lysed on ice for 30 min. The lysates were then centrifuged at 20,000 × g for 20 min at 4 °C to separate the pelleted (aggregated) proteins from the supernatant (soluble proteins). The resulting supernatants were mixed with SDS sample buffer, resolved by SDS‑PAGE, and transferred to PVDF membranes. Western blotting was performed using an anti‑KRAS G13D antibody. Band intensities were quantified by densitometric analysis, and the amount of soluble KRAS at each temperature was normalized to that at 37 °C (set as 1.0) to generate the thermal stability profile.

**Data S1**

| Supplementary table S1. Primer pairs used in this study | |
| --- | --- |
| GPX2-F | 5´-GATGACCCATTT TACCTCAT-3´ |
| GPX2-R | 5´-ATGTCAGGCTCAATGTTGAT-3´ |
| GAPDH-F | 5'-ACAGTCAGCCGCATCTTCTT-3' |
| GAPDH-R | 5'-GACAAGCTTCCCGTTCTCAG-3' |

**Data S2**

| Supplementary Table S2. Correlation between the Colorectal cancer features and expression of GPX2 | | | | |
| --- | --- | --- | --- | --- |
| **GPX2 expression** | | | | |
| **Clincopathological variables** | **Total** | **No. of low expression(%)** | **No. of high expression(%)** | ***P*-value** |
| **Gender** |  |  |  |  |
| Male | 182 | 59（32.42） | 123（67.580） | 0.8115 |
| Female | 124 | 46（37.09） | 78（62.91） |  |
| **Ages（years）** |  |  |  |  |
| ≤50 | 57 | 20（35.09） | 37（64.91） | 0.3387 |
| ＞50 | 249 | 95（38.15） | 154（61.85） |  |
| **Tumor size (cm)** |  |  |  |  |
| ≤5cm | 146 | 56（38.35） | 90（61.65） | 0.7505 |
| ＞5cm | 160 | 59（36.87） | 101（63.13） |  |
| **Tumor differentiation** |  |  |  |  |
| Well | 14 | 6（42.86） | 8（57.14） | 0.5683 |
| Moderate | 236 | 85（36.02） | 151（63.98） |  |
| Poor | 56 | 24（42.85） | 32（57.15） |  |
| **T stage** |  |  |  |  |
| T1 | 7 | 3（42.85） | 4 （57.15） | 0.0176 |
| T2 | 42 | 14（33.33） | 28 （66.67） |  |
| T3 | 93 | 39（41.93） | 54 （57.07） |  |
| T4 | 164 | 59（35.97） | 108（78.03） |  |
| **Lymph node involvement** |  |  |  |  |
| No | 161 | 55（34.16） | 106（65.84） | 0.0365 |
| Yes | 145 | 60（41.38） | 85（58.62） |  |
| **Distant metastasis** |  |  |  |  |
| No | 260 | 93（35.77） | 167（64.23） | 0.0364 |
| Yes | 46 | 21（45.65） | 25（54.34） |  |
| **Clinical stage** |  |  |  |  |
| Ⅰ+Ⅱ | 151 | 50（33.11） | 101（66.89） | 0.0097 |
| Ⅲ+Ⅳ | 155 | 65（41.93） | 90（58.06） |  |

**Data S3**

| Supplementary table S3. Sequences of siRNA used in this study | |
| --- | --- |
| GPX2 | Sense: 5′ CCCUCUGGUUGGUGAUUCATT 3′ |
